# aPPRove: An HMM-Based Method for Accurate Prediction of RNA-Pentatricopeptide Repeat Protein Binding Events

**DOI:** 10.1371/journal.pone.0160645

**Published:** 2016-08-25

**Authors:** Thomas Harrison, Jaime Ruiz, Daniel B. Sloan, Asa Ben-Hur, Christina Boucher

**Affiliations:** 1 Department of Computer Science, Colorado State University, Fort Collins, CO, 80523, United States of America; 2 Department of Computer and Information Science and Engineering, University of Florida, Gainesville, FL, 32611, United States of America; 3 Department of Biology, Colorado State University, Fort Collins, CO, 80523, United States of America; University of Cyprus, CYPRUS

## Abstract

Pentatricopeptide repeat containing proteins (PPRs) bind to RNA transcripts originating from mitochondria and plastids. There are two classes of PPR proteins. The P class contains tandem P-type motif sequences, and the PLS class contains alternating P, L and S type sequences. In this paper, we describe a novel tool that predicts PPR-RNA interaction; specifically, our method, which we call aPPRove, determines where and how a PLS-class PPR protein will bind to RNA when given a PPR and one or more RNA transcripts by using a combinatorial binding code for site specificity proposed by Barkan *et al.* Our results demonstrate that aPPRove successfully locates how and where a PPR protein belonging to the PLS class can bind to RNA. For each binding event it outputs the binding site, the amino-acid-nucleotide interaction, and its statistical significance. Furthermore, we show that our method can be used to predict binding events for PLS-class proteins using a known edit site and the statistical significance of aligning the PPR protein to that site. In particular, we use our method to make a conjecture regarding an interaction between CLB19 and the second intronic region of *ycf*3. The aPPRove web server can be found at www.cs.colostate.edu/~approve.

## 1 Introduction

Post-transcriptional control of RNA—which includes splicing, polyadenylation, and RNA editing—can have significant impact on the expression of a gene. One of the key factors that influences and contributes to post-transcriptional control of RNA is the availability and ability of specific proteins to bind to RNA. In short, RNA-binding proteins are those that bind to single- or double-stranded RNA and participate in forming ribonucleoprotein complexes. These complexes, in turn, exhibit a major role in post-transcriptional control of RNA [1, 2]. In this paper, we build a computational method for predicting where and how a family of RNA-binding proteins, the pentatricopeptide repeat (PPR), will bind to RNA. PPR proteins have generated significant interest and are well-known to have widespread existence in eukaryotes–in particular, land plants. Approximately 450 different PPR encoding genes have been found in *Arabidopsis thaliana* and rice (*Oryza sativa*). These proteins have vital interactions with RNA transcripts in mitochondria and plastids [3], have been demonstrated to be involved in RNA editing [4], and have shown to silence genes that encode for cytoplasmic male sterility (CMS) in flowering plants [5]. This latter role is of particular importance since male sterile plants are used to generate hybrid seed, which commercial agriculture heavily relies on for higher yield, and hence, highlights the interest of this class of proteins. Our method, which we call *aPPRove*, builds upon the recent work of Barkan *et al.* [6] that determines sequence-specific binding rules for PPR proteins.

The primary structure of many RNA-binding proteins—including PPR proteins—is composed of multiple repeats of a specific amino acid sequence, which recognize specific RNA sequences and/or structures [6–9]. We refer to the amino acid sequence where the RNA binds to as the *binding domain*, and the RNA sequence where the protein recognizes and binds to as the *binding site*. The length and the number of repetitions in the sequence corresponding to a binding domain varies widely across and within different classes of proteins [3]. Thus, there exists numerous computational methods that will determine and characterize the binding domains for a given RNA-binding protein, including HMMer [10], TPRPred [11], and ScanProsite [12]. The PPR family of proteins is classified by the existence of tandem PPR sequences, which are repeated any number of times [13]. These sequences compose the PPR proteins and are classified into three types based on the sequence length and composition: P-type sequences, which contain 35 amino acids, L-type sequences, which are slightly longer than P-type sequences, and S-type sequences, which are slightly shorter than P-type sequences. PPR proteins are classified into two classes based on the composition of PPR sequences: P-class proteins, which contain only tandem P-type sequences, and PLS-class proteins that contain alternating P, L and S type sequences. The PLS class of proteins are predominantly involved in C-to-U RNA editing [3, 4]. Given the primary structure of a PPR protein, we denote the sixth amino acid of a PPR sequence as *position* 6, and the first position of the next sequence as *position* 1′, thus using the same notation for these sites which was used in Barkan *et al.* [6]. Hence, if there exists *ℓ* repeated P, L and S type sequences in a PPR protein, then there are *ℓ* − 1 adjacent positions specified by positions 6 and 1′ in that protein. Fujii *et al.* [9] demonstrated that the amino acids at adjacent 6 and 1′ positions show site specificity. Barkan *et al.* [6] demonstrated that these two sites work in combination to bind to a nucleotide in an RNA transcript. Therefore, the sequence-specific relationship can be cast as an alignment problem where the question is how an RNA sequence aligns to two amino acid sequences (defined by the adjacent 6 and 1′ positions).

Our method takes as input a PPR protein and one or more RNA transcripts or RNA binding sites and outputs the binding domains that have highest statistical significance as well as how the nucleotides in the RNA are aligned to the amino acid pairs (defined by positions 6 and 1′) in these binding domains. First, the binding domain is identified using ScanProsite and the PROSITE database, and then, the extraction of the interacting residues is done using a tailored hidden Markov model (HMM) that aligns the RNA binding site to the binding domain. The transition and emission probabilities for the HMM are computed from existing data that describe known interactions between various classes of PPR proteins and their known RNA binding sites, a.k.a., the PPR-RNA bindings identified by Barkan *et al.* [6]. For each alignment, a p-value is computed by comparing against the scores derived from a large set of random alignments. Traditional motif detection is cast as the identification of statistically enriched patterns in a foreground set of sequences known to interact, and some background set of sequences expected to lack the binding site. The advantage of our method is that it does not require the identification of foreground and background sets, instead it leverages information about the binding specificities of PPR proteins already gleaned from previous work.

A typical use case of aPPRove is to determine how and where a PPR protein binds to a RNA transcript or binding site. We demonstrate that aPPRove can be used to predict putative binding sites in one or more RNA transcripts but there should be a prior belief that the PPR protein is known to target the transcript(s). In particular, our experiments show that each of the PPR-RNA binding events presented in Barkan *et al.* [6] have high statistical significance using cross validation which demonstrates the sensitivity and specificity of our approach. We show that aPPRove is capable of detecting putative binding events when presented with a PPR and RNA transcript that is deemed to have a binding site. We believe our method will be a useful tool for determining novel PPR-RNA binding events; rather than solely relying on laboratory techniques, aPPRove could be used to greatly narrow the search for novel binding events.

## 2 Related Work

The results of Barkan *et al.* [6] present a combinatorial binding code of PPR-RNA interaction that accounts for P and S motif sequences. They proposed a combinatorial binding code adhering to the rules shown in Table 1. This binding code was expanded by the findings of Yagi *et al.* [8] and Takenaka *et al.* [7] who discovered binding preferences of L-type sequences. Both found that a proline at position 6 of an L-type sequence is likely to bind to uracil. Furthermore, the results of Takenaka *et al.* [7] showed that asparagine at position 1′ of L-type sequences likely binds to adenine or uracil if it is paired with isoleucine, leucine, proline, threonine, or methionine at position 6. The model used in the three papers listed above involved aligning the PPR sequences of PLS proteins to the target RNA binding sites such that the terminal S-type sequence is positioned in contact with the nucleotide four base pairs upstream of an edit site on the target transcript. Okuda *et al.* [14] provides further evidence that PLS-class proteins align in this fashion. The pairing of positions 6 and 1′ in the PPR protein reinforced the previous findings of Fujii *et al.* [9]. Lastly, the results of Kotera *et al.* [4] demonstrated that PLS-class proteins are required for RNA editing.

**Table 1:** Demonstrating the combinatorial code for nucleotide specificity in Barkan *et. al* [6]. The first and second column contain the amino acid at site 6 and 1′ respectively. The third column contains the nucleotide the combination of the two amino acids show preference towards.

6	1′	Nucleotide Preference
T	D	G
S	N	A
T	N	A
N	D	U
N	N	C
N	S	C

Prior computational work in predicting protein-RNA interaction has focused on determining the actual binding site in the primary structure of the protein or the RNA sequence [10–12], developing protein-RNA interaction databases [15–17], and determining the likelihood that a particular protein will bind to an RNA [18–22].

The first computational method for predicting protein-mRNA interaction was proposed by Pancaldi and Bähler [18]. This method used Support Vector Machines (SVMs) and Random Forest (RF) classifiers to predict the likelihood of the interaction between a mRNA-binding protein and a mRNA. They used more than 1,000 features extracted from gene ontology terms, predicted secondary structures, mRNA properties, and genetic interactions. Two purely sequence-based approaches for predicting interaction likelihood were proposed by Muppirala *et al.* [20] and Wang *et al.* [21]. The method implemented in Muppiralla *et al.* [20] used RF and SVM classifiers to predict the probability of the interaction between a RNA-binding protein and RNA. It encoded the RNA sequences as normalized frequencies of tetrads. The protein sequences were encoded using a conjoined triad feature (CTF), and then used the amino acid composition and the nucleotide composition to predict the likelihood of one amino acid binding to a nucleotide. The method of Wang *et al.* [21] used a variation of CTF representation of protein descriptors and triads of the RNA sequence as RNA descriptors. These features were fed into both naïve Bayes and extended naïve Bayes classifiers.

A computational method specific to PPR-RNA interactions was presented in Yap *et al.* [22] where they predicted the recognition factor for an edit site on *atpF*. They aligned 6 and 1′ for 193 known PLS-class editing factors in such a way that the terminal S-type sequences aligned four base pairs upstream of the edit site and generated a score for each based on a table of log-likelihood ratios. Lastly, we note that all the methods predict the likelihood that a protein will bind to an RNA or mRNA molecule whereas aPPRove predicts how and where a PPR protein will bind to an mRNA using sequence-specific binding results.

## 3 Problem Formulation

The aim of aPPRove is to build a predictive model of PPR-RNA binding using sequence-specific binding rules. This can be cast as an alignment problem. Let *S*6 and *S*1′ be the amino acid sequences defined by position 6 and position 1′ of all adjacent motif sequences in the primary structure of a PPR protein *S*. If *S* contains *ℓ* adjacent motif sequences, *S*6 and *S*1′ both have length *ℓ* − 1. Hence, our problem is solved using a PPR protein *S*, an RNA transcript *R*, and a scoring function *ρ*. More formally,

$$\rho (S6_{i},S1_{j}^{\prime },R_{k}):aa\times aa\times N\to R,$$

where *N* = {*A*, *G*, *C*, *U*, −} and *aa* = {all possible amino acids and −}, where − signifies an insertion or deletion. The goal is to find the *w* top-scoring alignments between *R*, *S*6 and *S*1′ with respect to *ρ*. The following definition formalizes the problem that aPPRove solves.

**The Protein-RNA Sequence Binding Problem**

**Input:** An RNA sequence *R*, *S*6, *S*1′ and a scoring function *ρ*.

**Output:** The *w* top-scoring alignments of *R*, *S*6, and *S*1′.

## 4 Algorithms and Methods

aPPRove can be broken down into five main steps: (1) defining the repeat structure of the PPR by the motif sequence and number of repeats, (2) constructing *S*6 and *S*1′, (3) building a distribution of random alignments of *S*6 and *S*1′ to a database of RNA transcripts, (4) aligning *S*6 and *S*1′ to one or more RNA target transcripts, and (5) calculating the statistical significance (p-value) of the *w* top-scoring alignments of the PPR to target RNA transcripts.

### 4.1 PPR Motif Sequence Annotation

The PPR sequences are annotated using ScanProsite [23], a program that detects and annotates a protein sequence using the PROSITE database which contains signatures for various protein families and subfamilies; each signature is defined as a set of regular expressions or weight matrix [24]. ScanProsite is used with the PPR signature of the PROSITE database to identify the type of the PPR sequences in the protein. The PPR sub-class can be identified based on the type of the PPR sequences. PPR sequences containing fewer than 35 amino acids are assigned as a S-type, whereas those containing 35 amino acids are assigned as a P-type and those that do not fit in either of these classifications are assigned as a L-type [3]. After the PPR sequence annotation and type identification, *S*6 and *S*1′ are constructed from the two amino acids at position 6 and 1′ in each motif sequence, respectively, and *S*(6, 1′) are formed from the pairs of amino acids from *S*6 and *S*1′ of the same motif sequence. For example, if *S*1′ consists of *DDND*, and *S*6 consists of the set *SSTS*, then *S*(6, 1′) will be {(*DS*), (*DS*), (*NT*), (*DS*)}.

### 4.2 Alignment of a PPR Sequence to an RNA Target

**Fig 1:**
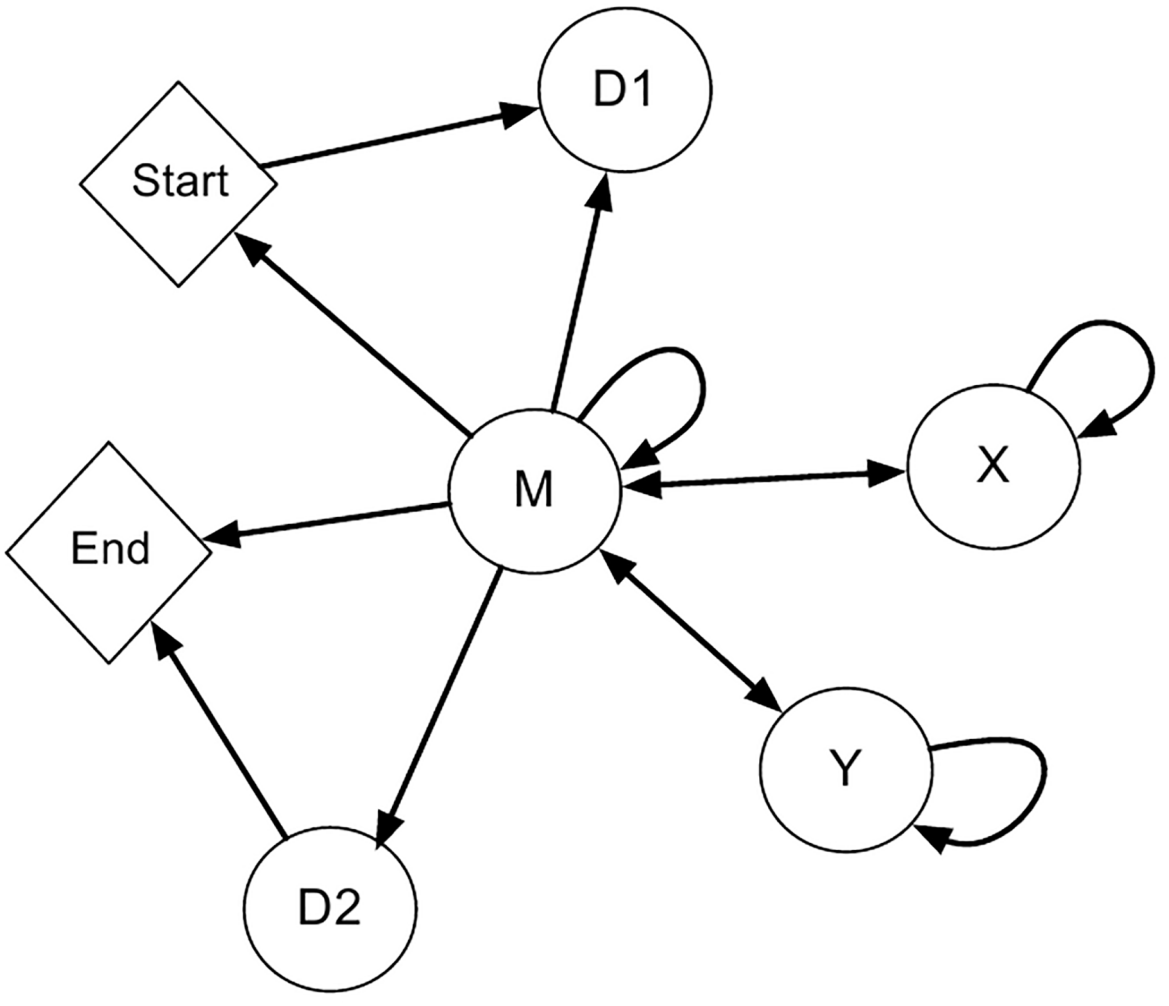
An Illustration of our paired HMM. Our model is tailored for semi-global alignment with seven states: *start*, *D*_1_, *D*_2_, *M*, *X*, *Y* and *end*. State *M* represents a match between an amino acid pair in *S*(6, 1′) and a target RNA nucleotide in an RNA transcript. States *D*_1_, *D*_2_ and *X* all represent a gap on the *S*6, 1′ side of the alignment. State *Y* represents a gap in the RNA sequence. *D*_1_ represents a gap in *S*(6, 1′) before the occurrence of a single match state and *D*_2_ represents a gap after all match states have occurred. State *X* represents a gap internal to *S*(6, 1′), meaning a state *M* should occur on both sides of any state *X*.

We use a paired HMM to align *S*(6, 1′) to a target RNA sequence (either a specific binding site or transcript). It is tailored for semi-global alignment with seven states: *start*, *D*_1_, *D*_2_, *M*, *X*, *Y*, and *end*, as shown in Fig 1.

State *M* represents a match between an amino acid pair in *S*(6, 1′) and a target RNA nucleotide. States *D*_1_, *D*_2_, and *X* all represent a gap in the *S*(6, 1′) side of the alignment. *D*_1_ represents a gap in *S*(6, 1′) before the occurrence of a single match state, and *D*_2_ represents a gap after all match states have occurred. State *X* represents a gap internal to *S*(6, 1′), meaning a state *M* should occur on both sides of any state *X*. Using separate states for the three different types of gaps in the alignment allow for different transition probabilities leaving from *D*_1_, *D*_2_, and *X*. Having these varying probabilities is necessary for semi-global alignment using a pair hidden Markov model. State *Y* represents a gap in the *R* side of the alignment.

We define a transition matrix **T** and emission matrices **A**, **O**, and **Q** in order to define our model. These matrices are constructed by using the binding events in figure S1 of Barkan *et al.* [6]; these binding events can be seen as alignments of the *S*(6, 1′) sequence of a PLS-class protein to the protein’s known RNA binding site. It is worth noting that these binding events —or alignments— are constructed by using previously identified binding rules. What results is a dataset that contains the frequency with which an amino acid pair binds to a specific nucleotide, as well as the frequency and location of insertions and deletions in the alignment. Hence, **T**, **A**, **O** and **Q** were defined using these data.

We now define some auxiliary variables that will be used for defining these matrices. First, we let *n* and *m* be equal to the length of *R* and *S*(6, 1′), respectively, and *F*(*α*, *β*) be the total number of times that state *α* transfers to state *β*, where *α* and *β* are in {*M*, *X*, *Y*, *D*_1_, *D*_2_, *end*, *start*}. For example, *F*(*M*, *X*) is equal to the number of times a gap follows a match in all the alignments obtained by Barkan *et al.* [6]. Let *G*(*i*, *j*, *k*) be equal to the total number times the *i*th amino acid pair is witnessed binding to nucleotide *j* in a *k*-type PPR sequence. Lastly, we let *γ* and *η* be a set of pseudo-counts used for determining the probabilities for **T** and **A**, respectively. We define *γ*(*i*, *j*) for all possible *i* and *j*, where *i* and *j* are states in the pair hidden Markov model. The variables *γ* and *η* are similarly defined.

The 6 × 6 transition matrix **T** defines the probability of transitioning from any one state to any other state. More formally, we define **T**(*α*, *β*) as the probability of state *α* transitioning to state *β*, where *α* is in {*start*, *M*, *X*, *Y*, *D*_1_, *D*_2_} and *β* is in {*M*, *X*, *Y*, *D*_1_, *D*_2_, *end*}. It should be noted that our model does not allow for transitioning from the *end* state or transitioning to the *start* state. The transition probability of leaving state *M* or *X* and transitioning to any other state, *i.e.* **T**(*M*, *β*) and **T**(*X*, *β*) where *β* is in {*M*, *X*, *Y*, *D*_1_, *D*_2_, *end*}, are defined according to the following formula:

$$\begin{array}{c} \frac{F(\alpha ,\beta )+\gamma (\alpha ,\beta )}{\sum_{\beta }\left(F(\alpha ,\;\beta )+\gamma (\alpha ,\;\beta )\right)}. \end{array}$$

The probabilities of transitioning from *start*, *D*_1_ and *D*_2_ and going to any other state are dependent on *n* and *m*. Hence, **T**(*D*_1_, *M*), **T**(*D*_2_, *end*), and **T**(*start*, *M*) are defined to be equal to 1/((*n*−*m*)/2). Next, we define **T**(*D*_1_, *D*_1_), **T**(*D*_2_, *D*_2_), and **T**(*start*, *D*_1_) as 1 − 1/((*n*−*m*)/2). We note that PLS-class proteins align in such a way that there will not be a transition to or from state *X* or state *Y*. This is because *S*(6, 1′) always aligns in a contiguous manner to its target site, as shown in Barkan *et al.* [6]. These two states were added for future flexibility in adapting the model for P-class proteins. Thus it can also be assumed that the length of *R* must be greater to or equal to the length of *S*(6, 1′).

Since there are 20^2^ possible amino acid pairs, four possible nucleotides, and three different types of PPR sequence, the emissions matrix **A** is of size 20^2^ × 4 × 3. The matrix **A** defines the emissions of state *M*. For example, A(IL,G,P) is the probability of witnessing the amino acid pair isoleucine (I) and leucine (L) binding to a guanine in a P-type sequence. The values for **A** were determined using the following formula:

$$\begin{array}{c} \frac{G(i,\;j,\;k)+\eta (i,\;j,\;k)}{\sum_{r}\sum_{q}\sum_{p}\left(G(p,\;q,\;r)+\eta (p,\;q,\;r)\right)}. \end{array}$$

The matrices **Q** and **O** have equal probability for all possible occurrences. Weighing all gap emission parameters evenly ensures that the algorithm will discriminate a good alignment based on the matches between statistically significant amino acid-nucleotide pairs as opposed to gaps.

We use the Viterbi algorithm for pair hidden Markov models [25] to find the optimal alignment score according to probabilities assigned to our transition and emission parameters. Let **VD**, **VM**, **VY**, and **VX** be four *n* × *m* dynamic programming matrices, where *n* is the number of pairs in *S*(6, 1′) and *m* is the length of *R*. The parameter *w* is provided as input by the user and causes the Viterbi algorithm to return the *w* top-scoring alignments according to the scoring scheme set by matrices **T**, **A**, **O**, and **Q**.

Upon the completion of the Viterbi algorithm **VD**, **VX**, **VY**, and **VM** contains scores for all sub-alignments ending in state *D*_2_, *X*, *Y*, and *M*, respectively. Every dynamic programming score is derived from the product of the score of the previous state, the probability of transitioning from the previous state, and the probability of the emission. The base case for this algorithm is as follows:

- Let **VD**(*i*, *j*)∧**VX**(*i*, *j*)∧**VY**(*i*, *j*)∧**VM**(*i*, *j*) = −∞:
  ∀(0 ≤ *i* ≤ *n*∧0 ≤ *j* ≤ *m*)
- Let **VD**(0, 0) = 1
- Let **VD**(*i*, 0) = **VD**(*i* − 1, 0) × **T**(*D*_1_, *D*_1_) × **Q**(*j*):
  ∀(0 < *i* ≤ *n*)

Matrices **VD**, **VX**, **VY**(*i*, *j*)∧ **VM** are completed with the following recurrence relation for ∀(0 < *i* ≤ *n*∧0 < *j* ≤ *m*).

$$\begin{array}{c} \text{VM}\left(i,\;j\right)=\text{the}\;w\;\max\left\{\begin{array}{cc} & \text{VD}\left(i-1,\;j-1\right)\times \text{T}\left(D_{1},\;M\right)\times \text{A}\left(i,\;j\right)\\ & \text{VM}(i-1,\;j-1)\times \text{T}(M,\;M)\times \text{A}(i,\;j)\\ & \text{VX}(i-1,\;j-1)\times \text{T}(X,\;M)\times \text{A}(i,\;j)\\ & \text{VX}(i-1,\;j-1)\times \text{T}(Y,\;M)\times \text{A}(i,\;j) \end{array}\right. \end{array}$$

$$\begin{array}{c} \text{VX}\left(i,\;j\right)=\text{the}\;w\;\max\left\{\begin{array}{cc} & \text{VM}(i-1,\;j)\times \text{T}(M,\;X)\times \text{Q}(j)\\ & \text{VX}(i-1,\;j)\times \text{T}(X,\;X)\times \text{Q}(j) \end{array}\right. \end{array}$$

$$\begin{array}{c} \text{VD}\left(i,\;j\right)=\text{the}\;w\;\max\left\{\begin{array}{cc} & \text{VM}\left(i-1,\;j\right)\times \text{T}\left(M,\;D_{2}\right)\times \text{Q}\left(j\right)\\ & \text{VD}\left(i-1,\;j\right)\times \text{T}\left(D_{2},\;D_{2}\right)\times \text{Q}\left(j\right) \end{array}\right. \end{array}$$

$$\begin{array}{c} \text{VY}\left(i,\;j\right)=\text{the}\;w\;\max\left\{\begin{array}{cc} & \text{VM}(i,\;j-1)\times \text{T}(M,\;Y)\times \text{O}(i)\\ & \text{VX}(i,\;j-1)\times \text{T}(Y,\;Y)\times \text{O}(i) \end{array}\right. \end{array}$$

The scores of the *w* top-scoring alignments are found at **VD**(*n*, *m*) and **VM**(*n*, *m*). Traditional Viterbi decoding is used to obtain the sequence of states and hence the alignment associated with each of the *w* highest scores. Each of the *w* optimal scores is normalized by summing up all transition and emission probabilities that correspond to transitioning to a state *M* or *X*, subtracting **T**(*D*_1_, *M*) from this total, and dividing this score by the length of the sub-alignment.

### 4.3 Statistical Significance of Scores

aPPRove returns a p-value for each of the *w* top-scoring alignments; this p-value statistic describes the probability of obtaining a normalized score that is at least as significant as the one that was actually observed. In order to calculate p-values, we require a database of possible alignments. By default, aPPRove considers all possible bindings to a database of plastid *Arabidopsis thaliana* transcripts. The set of *Arabidopsis thaliana* transcripts was obtained from the Phytozome website V9, which can be accessed at: http://phytozome.jgi.doe.gov/pz/portal.html. By default, we align *S*(6, 1′) to each possible location in every transcript in the database and the targeted RNA transcripts for the given PPR sequence, which results in a normalized score of every position of every alignment (either to the RNA in the database or the targeted RNA). These scores are normally distributed. Normality of these distributions were determined empirically and are shown in S1 Fig. Thus, the p-value is calculated using the null hypothesis that the normalized score is equal to the mean of the distribution.

aPPRove uses the *Arabidopsis thaliana* plastid transcripts by default; however, any user-defined database of RNA transcripts can be specified. If run with a custom database, aPPRove will provide the p-values of the *w* highest normalized scores by using the normal distribution of normalized scores of aligning *S* to the database. In addition, it is possible to run aPPRove without using any database (default or otherwise). In this case, aPPRove outputs the normalized scores of the *w* top-scoring alignments and the details of the alignments but no p-values.

## 5 Results

### 5.1 Data

We used the dataset from figure S1 of Barkan *et al.* [6] to parameterize and evaluate the performance of our model. All the data is freely available from the website. This dataset is composed of 30 PLS-class proteins and their known binding site RNA sequences. Because some proteins bind to multiple targets, there is a total of 55 instances of a PPR protein paired with a known binding site. All of these proteins target transcripts originating from either mitochondria or plastids. Of the 30 proteins, 27 are from *Arabidopsis thaliana*, two are from moss (*Physcomitrella patens*), and one is from rice (*Oryza sativa*). Protein sequences from this dataset were extracted from either GenBank [26] or Uniprot [27]. The names and accession numbers of these editing numbers are available in the Software and Data Availability section. However, PpPPR56, PpPPR71, PpPPR78 and PpPPR79 were not used for the evaluation because they were only available as sequence fragments. Additionaly, PPR2263 was not used because it is only available as a hypothetical sequence, and MEF14 was not used because we were not able to find PPR sequences using PrositeScan. Given that PPR protein domains could have more than one binding site, there were a total of 55 protein domain and binding site pairings, with 45 of these pairing involving *Arabidopsis thaliana* domains, seven pairings involving *Oryza sativa* domains and three pairings involving *Physcomitrella Patens* domains.

### 5.2 Statistical Analysis of Aligning Proteins to Their Target Sites

**Fig 2:**
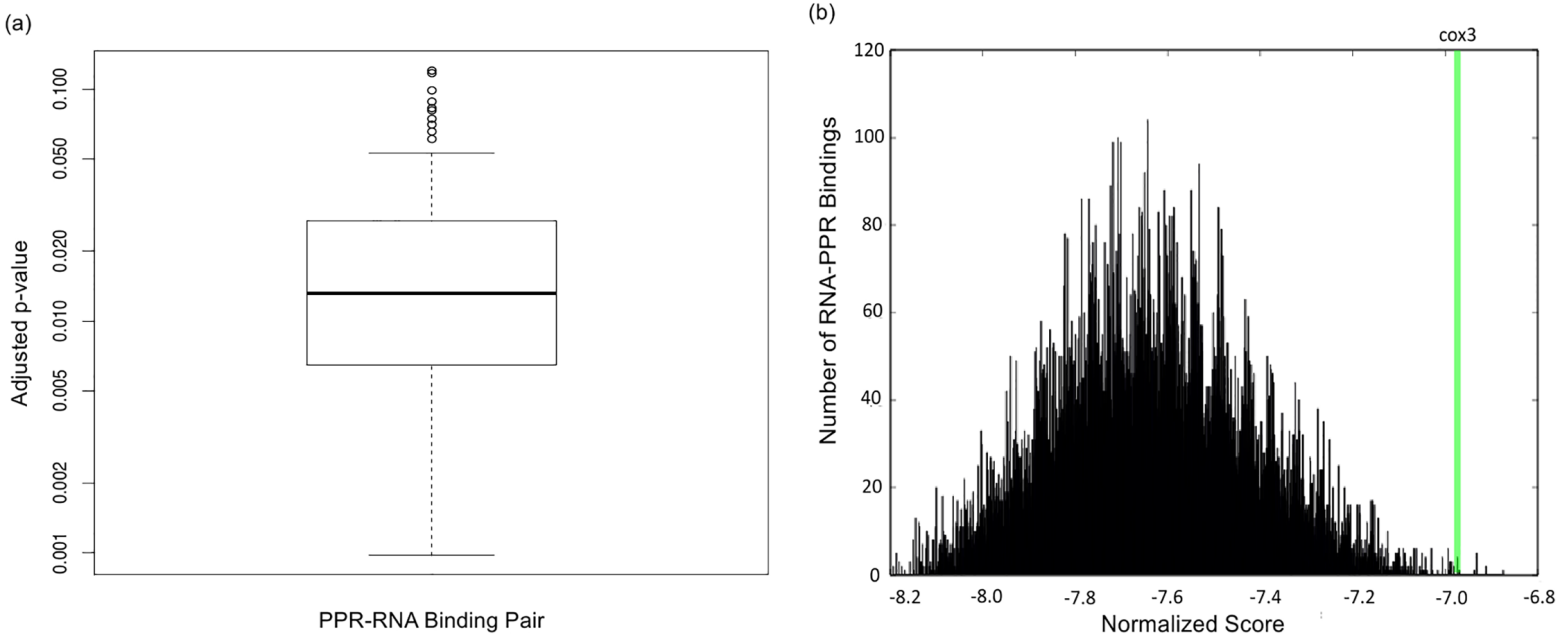
An illustration of the distribution of normalized scores and the corresponding adjusted p-values. Fig 2(a) is a boxplot of the 55 Benjamini Hochberg adjusted p-values of the normalized scores. The median p-value is 0.013. Fig 2(b) illustrates the distribution of normalized scores, which are calculated by finding all possible alignments of every PLS protein to each possible binding site in the target database (including the known binding site) and then normalizing all of these scores. The normalized score of the known binding should have a relatively lower adjusted p-value and thus, be identified in the extreme right of the distribution. The green line indicates where the score of aligning MEF26 to its known binding site on *cox*3 is located on the distribution generated by aligning the *S*(6, 1′) sequence of MEF26 to the target database [29].

In order to determine the statistical significance of a PPR protein domain binding to its known binding site, we compared the score of aligning *S*(6, 1′) of each PPR protein to its own binding site against every possible contiguous alignment of *S*(6, 1′) to a database of transcripts. Two databases were used for this investigation. One database consisted of all transcripts from the *Arabidopsis thaliana* plastid, and the other consisted of all transcripts from the *Arabidopsis thaliana* mitochondrion. We selected the database to use for each run based on what type of organelle transcripts that particular protein targets. We evaluated our method by using Leave One Out cross validation (LOO) for each PPR binding domain and RNA binding site. Thus, for each pair, we parametrized the paired HMM using all other pairs except the one being evaluated, ran the trained model on the pair that was removed, and determined the normalized score for the pair of interest. Using the transcript database, a p-value for each PPR binding domain and RNA binding site pair was found using its normalized score and then adjusted using the Benjamini Hochberg method [28]. As shown in Fig 2, the median of all 55 p-values is 0.013.

Also shown in Fig 2, there exists two adjusted p-values greater than 0.1, which correspond to the MEF1-*nad*2 binding and the CRR28-*ndhD* binding. The larger p-value of these bindings is unsurprising because the corresponding alignments are such that the these two proteins align to their target sites in such a way that amino acids pairs with high site specificity are not paired with their preferred nucleotide [6]. Two out of the six amino acid pairs have high site specificity in the alignment of CRR28 to *ndhD* and three out of the six amino acid pairs have high site specificity in the alignment of MEF1 to *nad*2. Thus, this experiment validates our approach and demonstrates that the known PPR and RNA binding pairs can be identified by considering the extreme values of the distribution.

**Fig 3:**
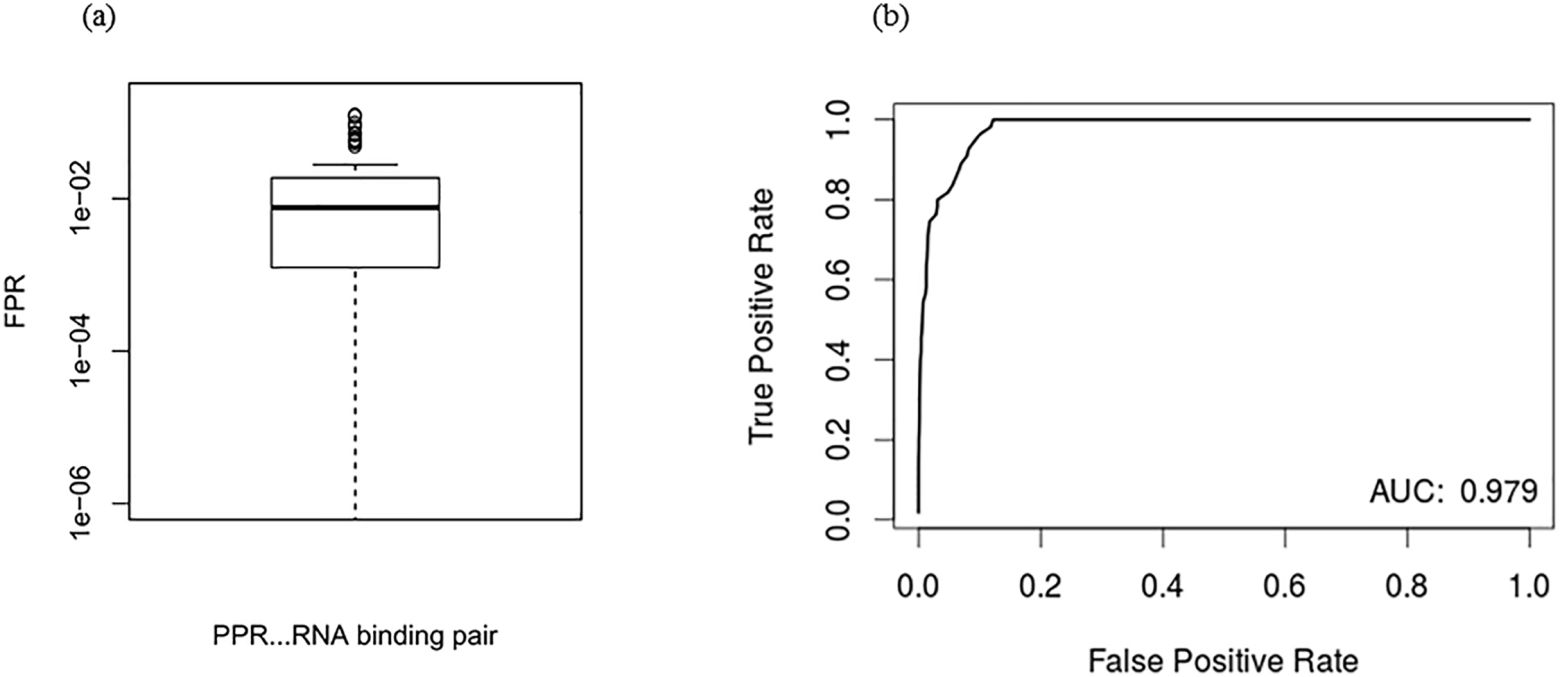
An illustration demonstrating the relationship between the True Positive Rate (TPR) and False Positive Rate (FPR) of the aPPRove algorithm. (a) illustrates the FPR of all 55 PPR-RNA pairs. We compared the score of aligning *S*(6, 1′) of each PPR protein to their own binding site against every possible alignment to a database of decoy transcripts. The median and range of the FPR is 0.0076 and 0.12. (b) is the ROC curve that was computed by running aPPRove on all 55 PPR proteins and their known binding sites, using the set of *Arabidopsis thaliana* transcripts originating from the organelle that the PPR targets.

To find the false positive rate (FPR) for each of the 55 PPR and RNA binding site pairs, we compared the score of aligning *S*(6, 1′) of each PPR protein to its known binding site against every possible contiguous alignment of *S*(6, 1′) to a database of decoy transcripts. The two decoy databases were created by generating a random permutation for each transcript from the target database. Similar to the analysis that used the target databases, we evaluated our method by using LOO for each PPR and binding site pair. For each pair the FPR was calculated by the ratio of the number of alignments to the decoy database that had a normalized score greater than or equal to the score of aligning the PPR to its binding site over the total number of alignments to the decoy database. Fig 3(a) illustrates the median and range of the FPR. In particular, the median and range of the FPR are 0.0076 and 0.12, respectively.

A receiving operator characteristic (ROC) curve was constructed in order to view the sensitivity and specificity of the aPPRove algorithm. To construct this curve, aPPRove was ran on all 55 PPR proteins and their known binding site, using the set of *Arabidopsis thaliana* transcripts originating from the organelle that the PPR targets. Again, the normalized scores were calculated using LOO cross validation. All 55 p-values of aligning a PPR protein to its binding site were considered positive instances. We took the p-value of the normalized score of every possible alignment within its own distribution, and pooled the p-values of all 55 distributions together. These instances were considered negative. Fig 3(b) shows the ROC curve built by this experiment. The area under the curve (AUC) is 0.979, demonstrating that most positives will be classified as true positive using a low discrimination threshold for all instances.

### 5.3 Binding Event Prediction Using Previously Discovered Edit Sites

Site-specific RNA editing factors continue to be discovered at a rapid rate, including many that have been identified since the dataset that we used to train our model was compiled [6]. For example, Arenas-M. *et al.* [29] demonstrated in the absence of MEF26, *cox*3-311 editing is completely abolished and *nad*4-166 is only partially edited. Using aPPRove, we confirmed that the two predicted binding sites with the alignment ending four base pairs upstream of the two edit sites were both among the top 41 hits out of 66,500 total number of possible alignments in the mitochondrial target database. Both of these have a p-value less than 0.0005.

**Fig 4:**
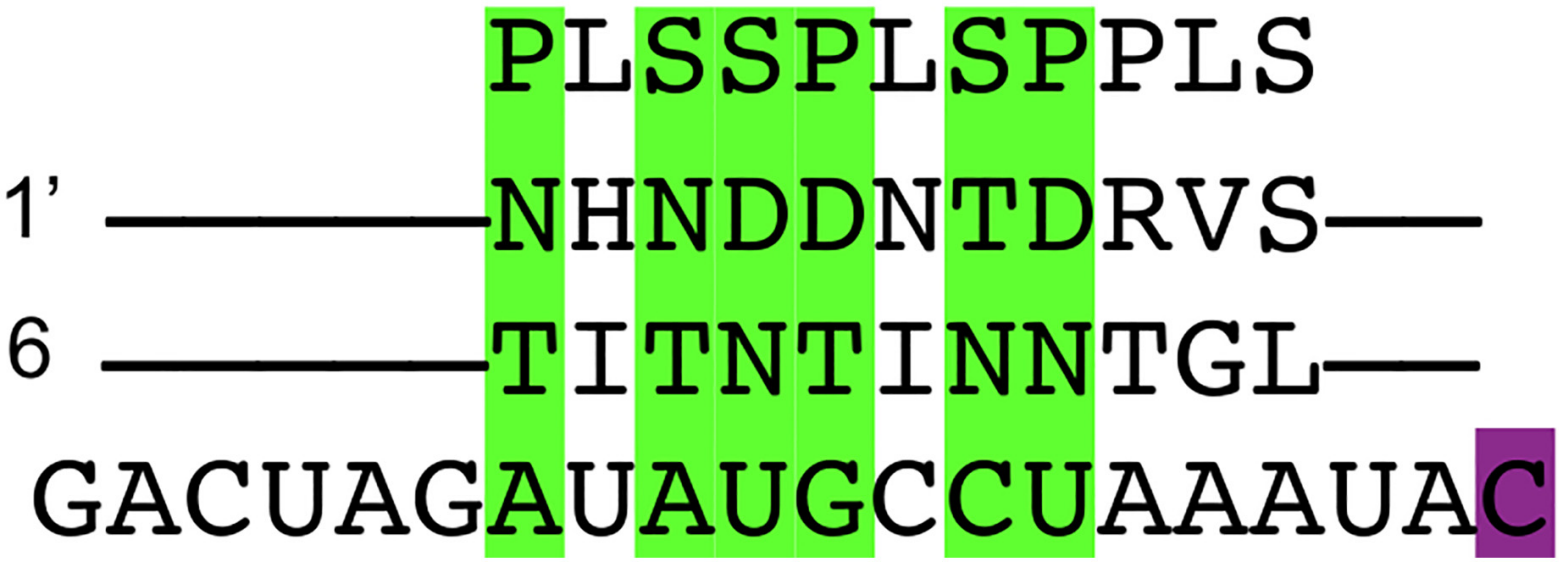
An illustration of the putative binding event of CLB19 and *ycf3*. This shows the alignment of the putative binding event of CLB19 and the binding site located upstream of the edit site at position 43,350 of the *Arabidopsis thaliana* plastid genome. Pairs highlighted in green are considered to be statistically correlated amino acid-nucleotide pairs as specified by Barkan *et al.* [6]. The C highlighted in magenta is the edit site of the binding site.

We aligned the 12 PPR proteins known to target the *Arabidopsis thaliana* plastid from our data set to one of the nine minor binding sites [30] found at genomic position 43,350 located in the second intronic region *ycf*3. This particular binding site was selected at random. We sampled the sequence 30 base pairs upstream of the edit site and aligned all 12 PPR proteins to it. Of these proteins, CLB19 had the lowest p-value at 0.00005 and aligned to this target site in such a way that all six amino acid pairs with high site specificity aligned to their preferred nucleotide. Given the low p-value as well as the distance from the edit site, we predict that CLB19 is the editing factor for this edit site. Fig 4 illustrates this predicted putative binding event.

### 5.4 Factors Influencing the Predictive Ability of aPPRove

**Fig 5:**
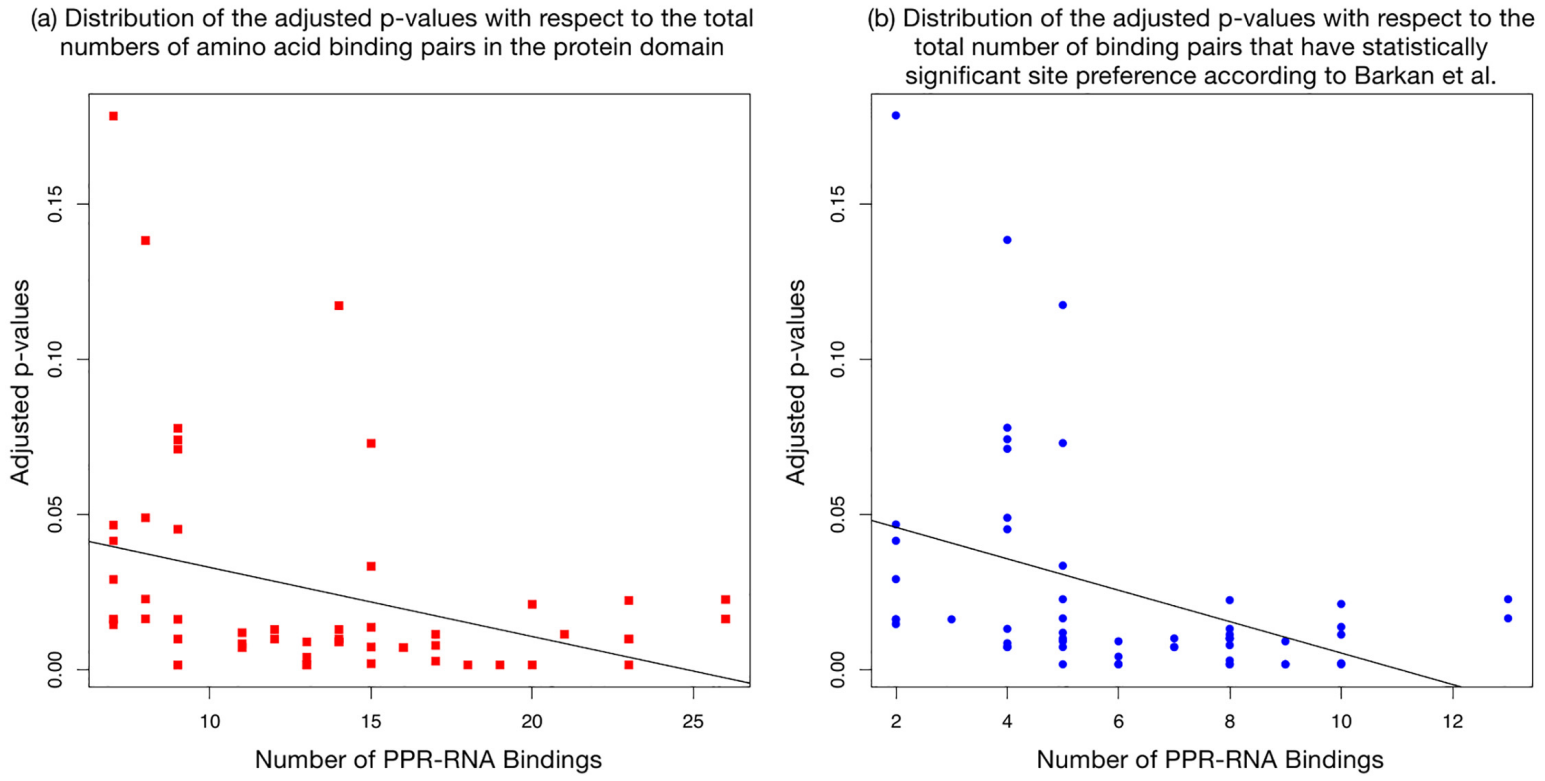
Illustrations that demonstrate the adjusted p-values with respect to the total number of amino acid binding pairs in the protein domain, and the adjusted p-values with respect to the total number of binding pairs that have statistically significant site preference according to Barkan *et al.* [6]. The regression lines on both plots demonstrate that there is an negative correlation between the number of binding pairs in the protein domain and p-value. (a) has a Pearson’s Correlation sample estimate of −0.335024 with a p-value of 0.01241. (b) has a Pearson’s Correlation sample estimate of −0.3978517 with a p-value of 0.00263.

We note that aPPRove is more successful in predicting the binding of PPR proteins with a larger number of PPR sequences than proteins with a fewer numbers since those with a fewer number result in more false positives because there are fewer amino acid pairs to show preference to the nucleotides in *S*(6, 1′). Fig 5 demonstrates the adjusted p-values with respect to the total numbers of amino acid binding pairs in the protein domain as well as the total number of binding pairs that have statistically significant site preference according to Barkan *et al.* [6]. The regression lines in Fig 5 demonstrate that there is a negative correlation between the number of amino acid binding pairs in a binding and the p-value of the binding.

Thus, the results indicate that aPPRove can locate how and where binding PLS-class of PPR proteins will bind to their target transcript and provide the statistical significance of a particular binding. Lastly, we showed the binding prediction of aPPRove increases as the length of the *S*(6, 1′) increases.

### 5.5 Practical Considerations: Memory and Time

We evaluated the memory and time requirements of aPPRove. Since aPPRove is a multi-threaded application, its wall-clock time depends on the computing resources available to the user. aPPRove required a maximum of 12 threads, 1 gigabyte of RAM and 24 hours for all previously described experiments. In addition to these experiments, we used aPPRove with the *Arabidopsis thaliana* a database of 88 plastid transcripts to predict the top-scoring alignment to the transcriptome. In order to accomplish this experiment, we downloaded a file of all TAIR10 cDNA sequences with 5’ and 3’ UTRs.This file consisted of 41,671 transcripts totalling approximately 66 × 10^6^ base pairs. This experiment completed under 24 hours using 12 threads and 1 gigabyte of RAM. However, we note that the web interface does email the results to the requested address and therefore, does not require active engagement during the run time of the software.

## 6 Conclusion

We presented a method that used the primary binding code of PPR proteins to predict how a protein will bind to a target transcript or binding footprint. Our method is unique in that it can be used to detect where and how a PPR protein binds to an RNA as opposed to assessing the likelihood of interaction. Again, we note that the hidden Markov model was parametrized with a dataset involving protein-RNA interactions of only PLS-class proteins, thus aPPRove captures the intricacies of how the PLS class of PPR proteins bind to their target, but it may not accurately portray how a P-class PPR protein will bind to its target. The lack of data regarding P-class PPR protein interactions prevents us from adapting the model specifically for this subfamily of proteins. It is possible that the onset of high throughput methods of quantifying protein-RNA interactions [31] may allow for future progress in modeling the interaction of P-class proteins and their target transcripts. Finally, if there is a known edit site, aPPRove can be used to detect putative binding events. Detecting these events is one of the most beneficial and powerful uses of aPPRove.

Lastly, we note that the data used for our investigation was compiled from a number of experimental techniques that are not high throughput. One commonly used technique is a gel mobility shift assay. This involves mixing the RNA-binding protein with a short RNA sequence and running the sample through a gel. If the RNA is bound to the protein, it will run slower because of the larger size. If not, it will quickly run through the gel. Using this technique allows for the separation of bound and unbound RNA molecules. Performing this experiment on many different RNAs can narrow down the necessary window for binding. Prikryla *et al.* [32] demonstrated other methods that are specific to PPR proteins. Although these techniques are not high-throughput, there is evidence that such methods are on the horizon. In 2014, Tome *et al.* [31] developed a high-throughput sequencing-RNA affinity profiling assay by adapting a high-throughput genome sequencer to quantify the binding of a protein to millions of RNAs. As high-throughput methods become more commonplace, greater numbers of datasets that are larger will become available. aPPRove is one method that can be easily adapted with forthcoming data and thus be used to predict the binding of other families and subfamilies of proteins.

## Software and Data Availability

Software and data can be accessed from the aPPRove web page (www.cs.colostate.edu/~approve) or from our github repository: https://github.com/approve-molbio/aPPRove. The data include edit sites and fasta files for the following editing factors: CLB19 (AEE27887.1), CRR21 (NP_200385.1), CRR22 (NP_172596.1), CRR28 (NP_176180.1), CRR4 (NP_182060.2), LPA66 (AED95742.1), MEF1 (AED96243.1), MEF11 (AEE83509.1), MEF14 (Q9LW33), MEF18 (AED92640.1), MEF19 (AEE74210.1), MEF21 (AEC07025.1), MEF22 (AEE75244.1), MEF26 (Q9SS60), MEF29 (Q9SUH6), MEF3 (Q9LND4), MEF7 (Q9FIB2.1), MEF9 (O04590), OGR1 (ACL79585.1), OTP80 (AED97156.1), OTP81 (AEC08301.1), OTP82 (AEE28239.1), OTP84 (Q7Y211), OTP85 (AEC05651.1), OTP87 (NP_177599.1), PpPPR_77 (BAD67156.2), PpPPR_91 (BAD67154.1), RARE1 (AED91873.1), REME1 (NP_178481.1), SLG1 (Q9FNN9), SLO1 (Q9SJZ3), YS1 (F4J1L5).

## Supporting Information

S1 Fig Distributions of Normalized Scores of Aligning *S*(6, 1′) of a PPR to Each Possible Location in to every Transcript in the Database. Distributions were built for 55 protein domain and binding site pairings. We selected the database to use for each run based on what type of organelle transcripts that particular protein targets. We evaluated our method by using Leave One Out cross validation (LOO) for each PPR binding domain and RNA binding site.
(PDF)
