# Supplementary material for: aPPRove: An HMM-Based Method for Accurate Prediction of RNA-Pentatricopeptide Repeat Protein Binding Events
Source: PLoS One. 2016 Aug 25;11(8):e0160645. doi: 10.1371/journal.pone.0160645 (PMC4999063; doi:10.1371/journal.pone.0160645)
Supplement: S1 Fig — Distributions were built for 55 protein domain and binding site pairings. We selected the database to use for each run based on what type of organelle transcripts that particular protein targets. We evaluated our method by using Leave One Out cross validation (LOO) for each PPR binding domain and RNA binding site. (PDF) [file pone.0160645.s001.pdf]

Distributions of Normalized Scores of Every Alignment to the Target Database of Each Protein and It's Target Site

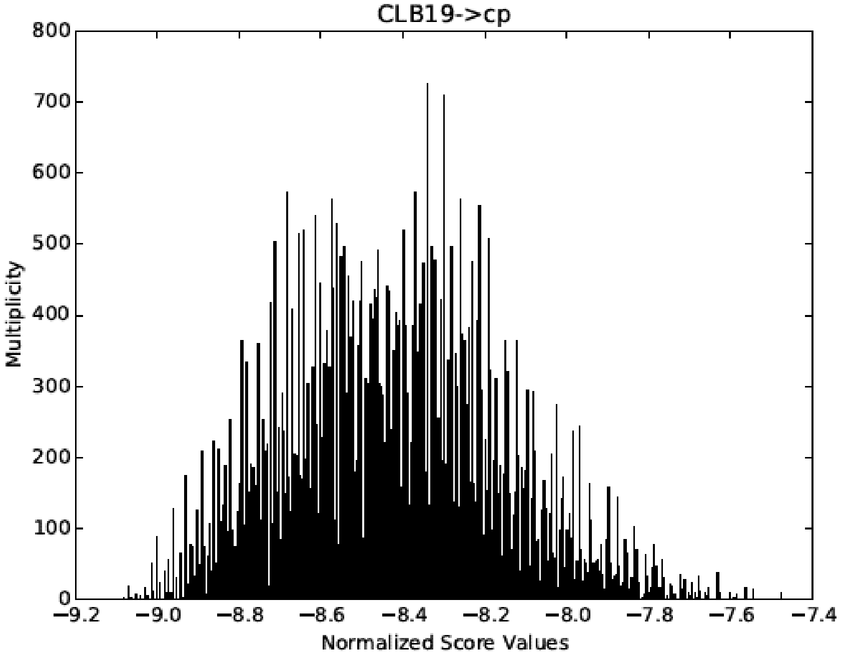

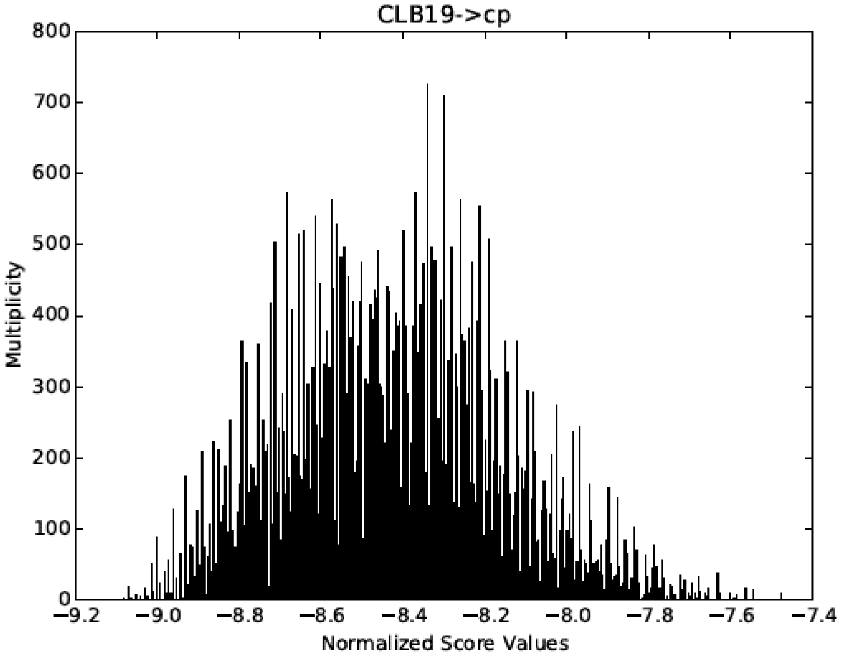

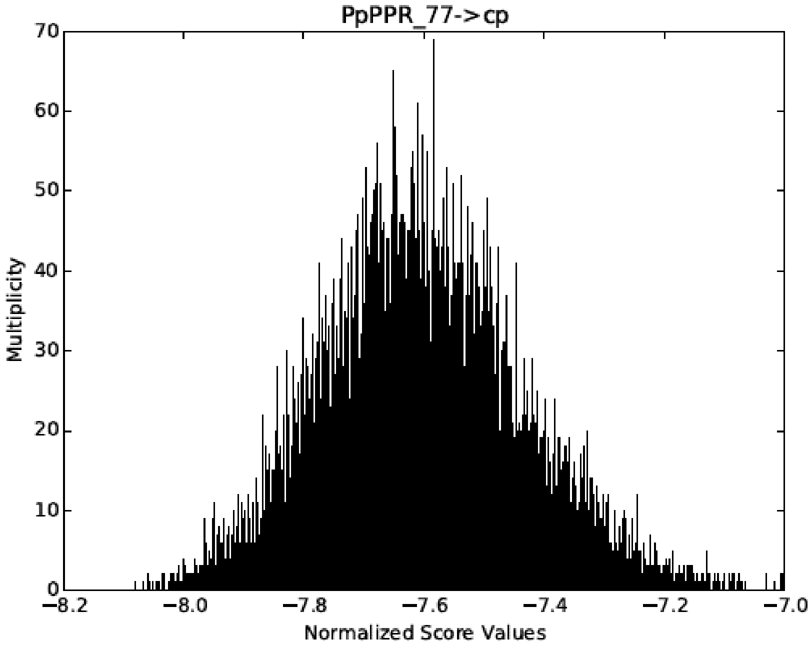
PpPPR_77->cp
Multiplicity
Normalized Score Values
0
10
20
30
40
50
60
70
−8.2
−8.0
−7.8
−7.6
−7.4
−7.2
−7.0

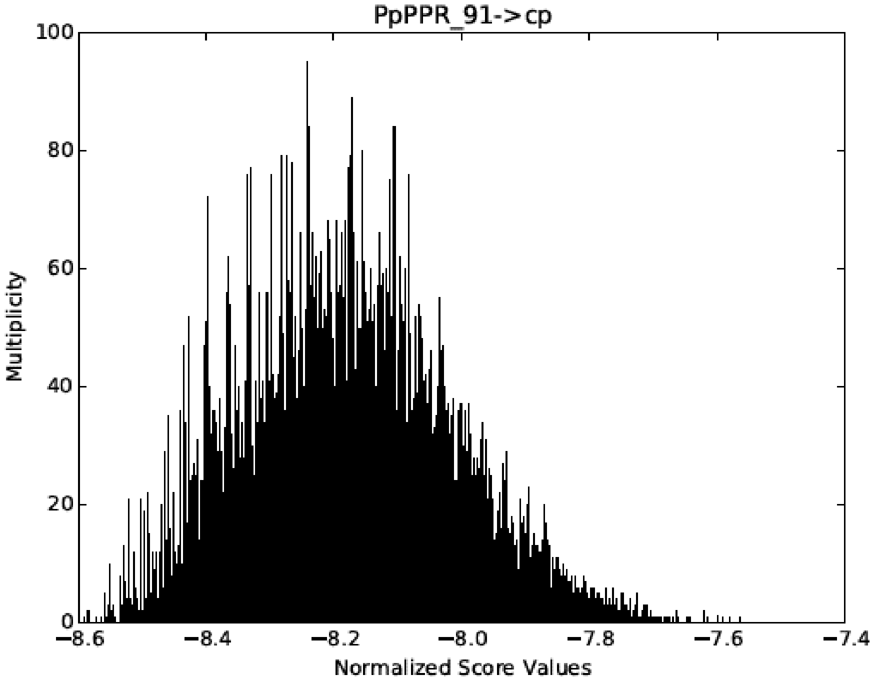
PpPPR_91->cp
Multiplicity
Normalized Score Values
0
20
40
60
80
100
−8.6
−8.4
−8.2
−8.0
−7.8
−7.6
−7.4

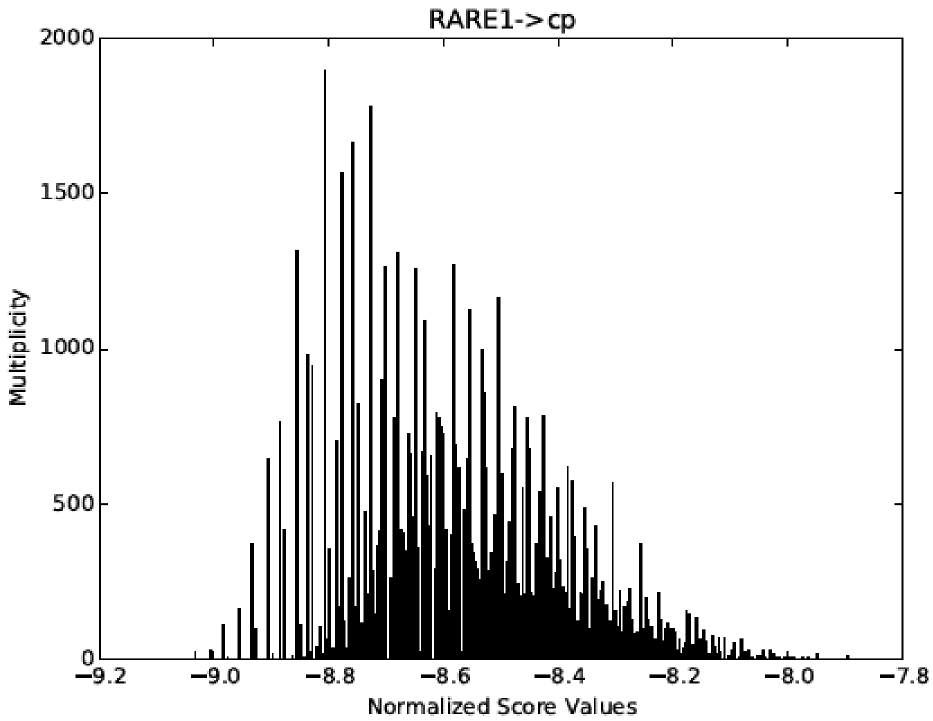
RARE1->cp
Multiplicity
Normalized Score Values
2000
1500
1000
500
0
−9.2
−9.0
−8.8
−8.6
−8.4
−8.2
−8.0
−7.8

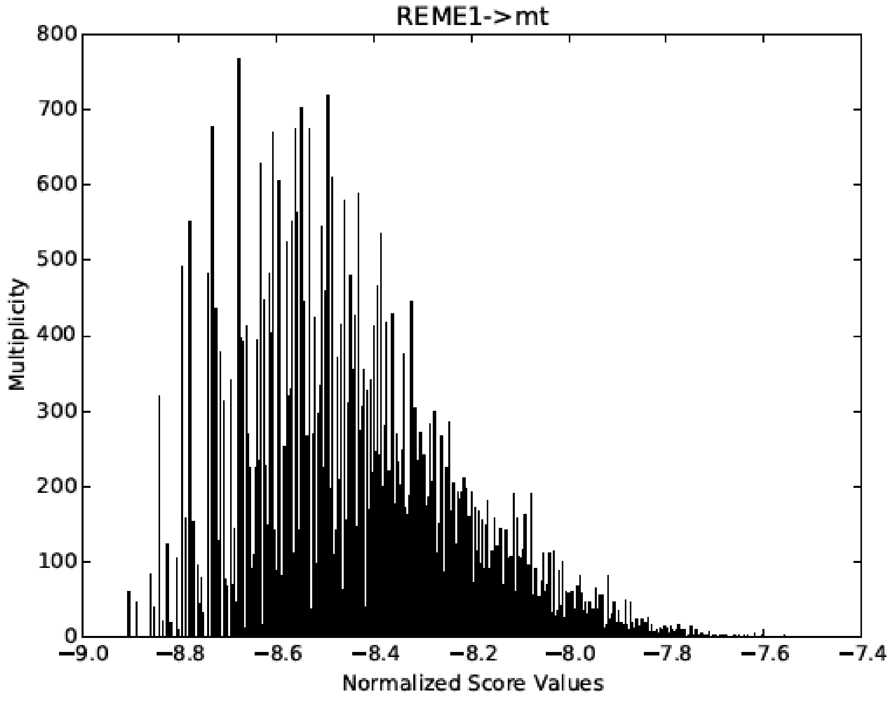
REME1->mt
Multiplicity
Normalized Score Values
800
700
600
500
400
300
200
100
0
−9.0
−8.8
−8.6
−8.4
−8.2
−8.0
−7.8
−7.6
−7.4

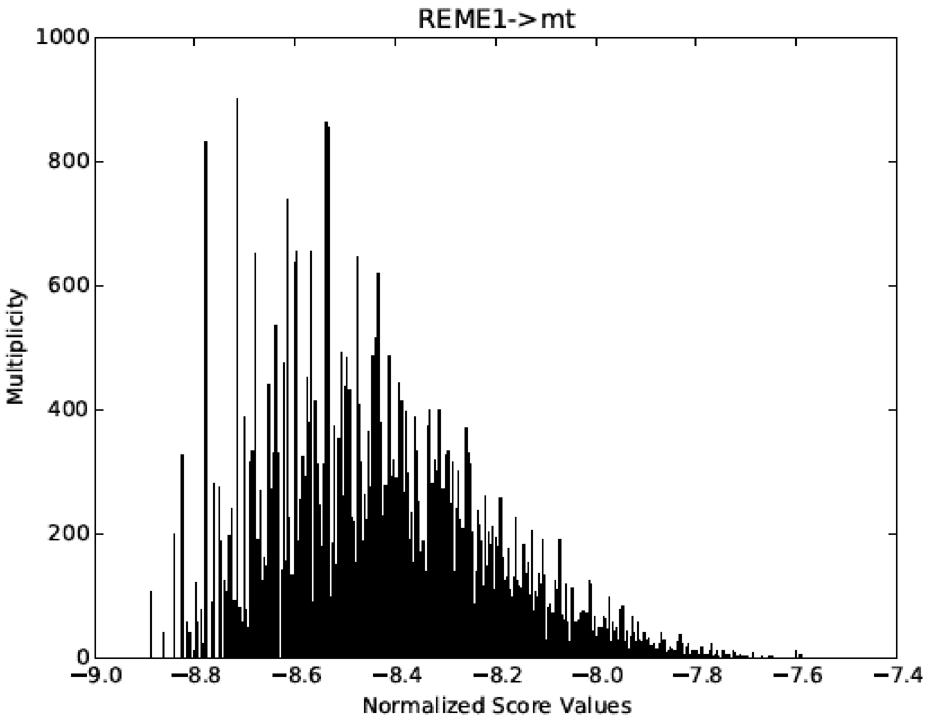

REME1->mt
Multiplicity
Normalized Score Values
0
200
400
600
800
1000
−9.0
−8.8
−8.6
−8.4
−8.2
−8.0
−7.8
−7.6
−7.4

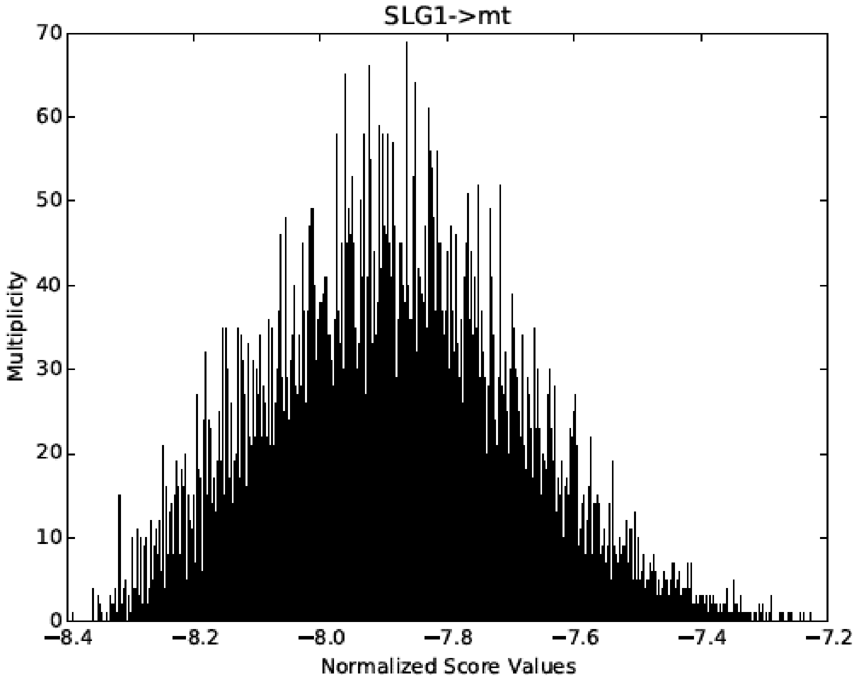

SLG1->mt
Multiplicity
Normalized Score Values
0
10
20
30
40
50
60
70
−8.4
−8.2
−8.0
−7.8
−7.6
−7.4
−7.2

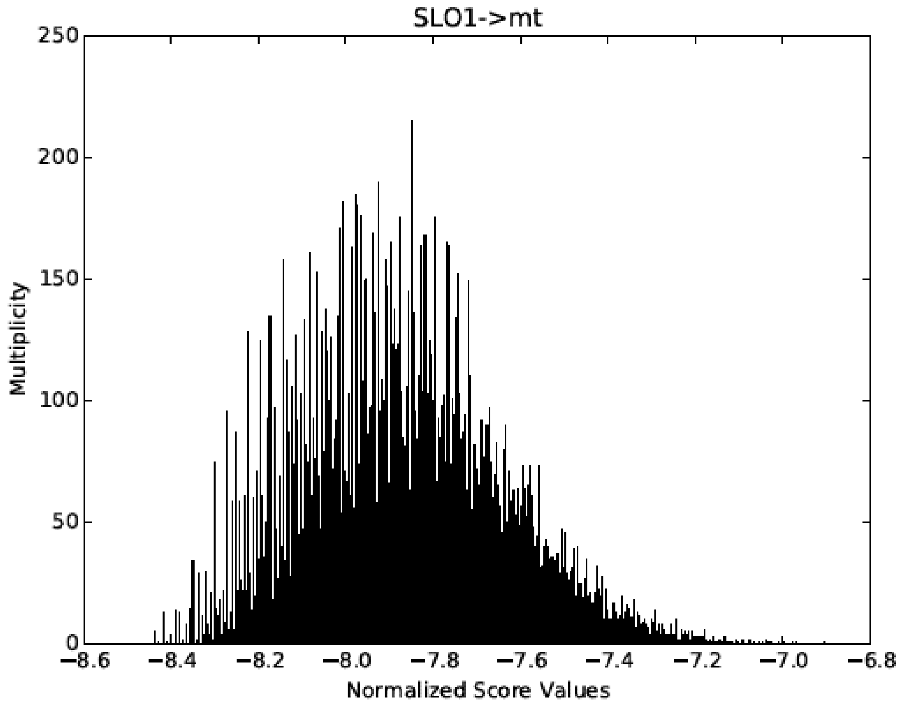
SLO1->mt
Multiplicity
Normalized Score Values
250
200
150
100
50
0
−8.6
−8.4
−8.2
−8.0
−7.8
−7.6
−7.4
−7.2
−7.0
−6.8

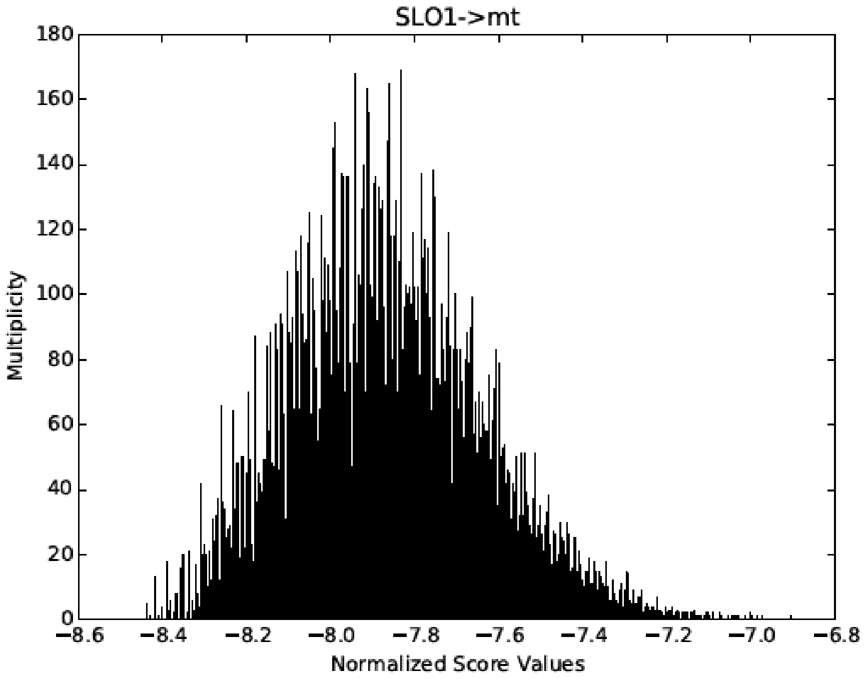
SLO1->mt
Multiplicity
Normalized Score Values
180
160
140
120
100
80
60
40
20
0
−8.6
−8.4
−8.2
−8.0
−7.8
−7.6
−7.4
−7.2
−7.0
−6.8

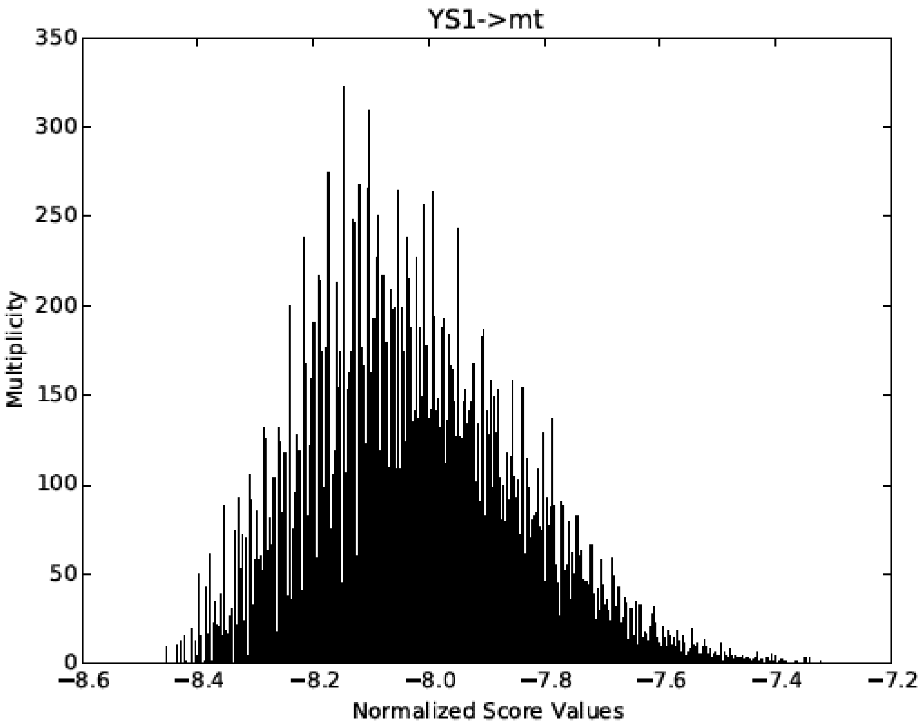
YS1->mt
Multiplicity
Normalized Score Values
350
300
250
200
150
100
50
0
−8.6
−8.4
−8.2
−8.0
−7.8
−7.6
−7.4
−7.2

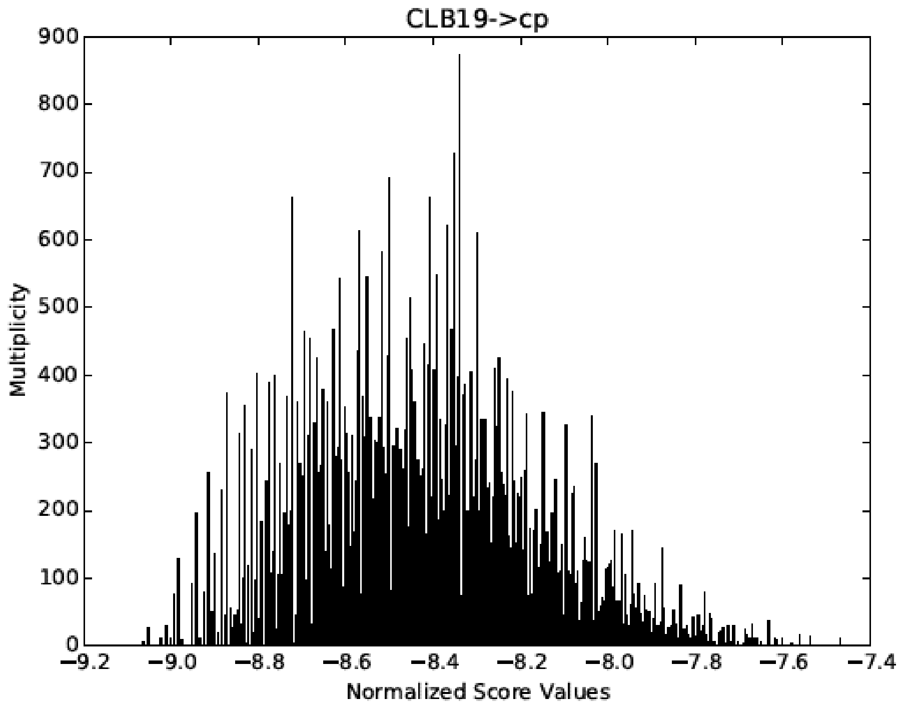
CLB19->cp
Multiplicity
Normalized Score Values
900
800
700
600
500
400
300
200
100
0
−9.2
−9.0
−8.8
−8.6
−8.4
−8.2
−8.0
−7.8
−7.6
−7.4

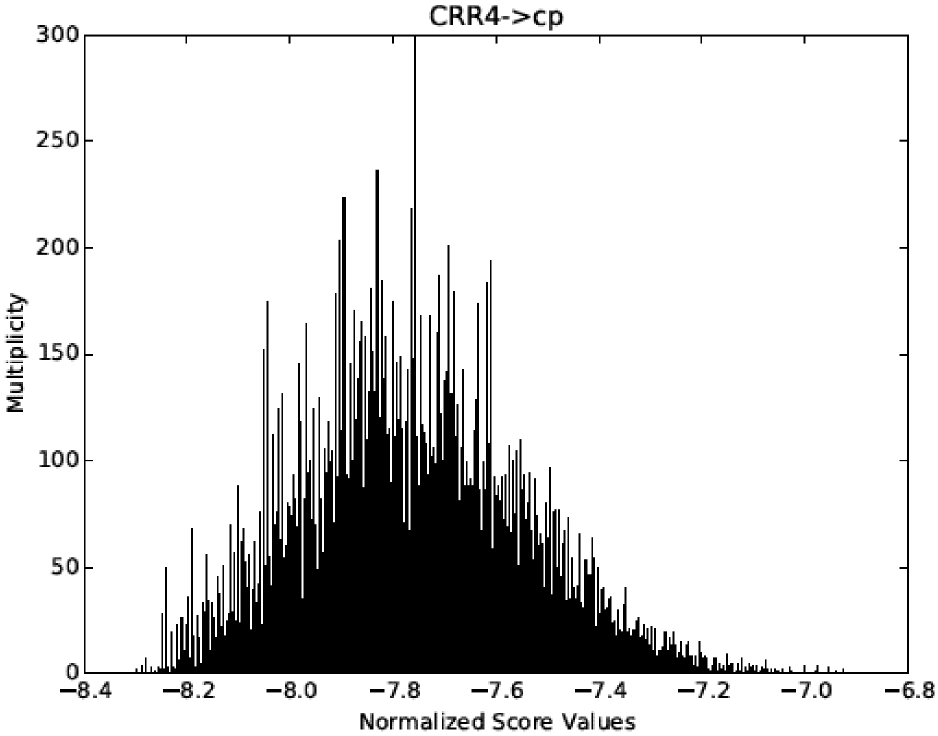

CRR4->cp
Multiplicity
Normalized Score Values
300
250
200
150
100
50
0
−8.4
−8.2
−8.0
−7.8
−7.6
−7.4
−7.2
−7.0
−6.8

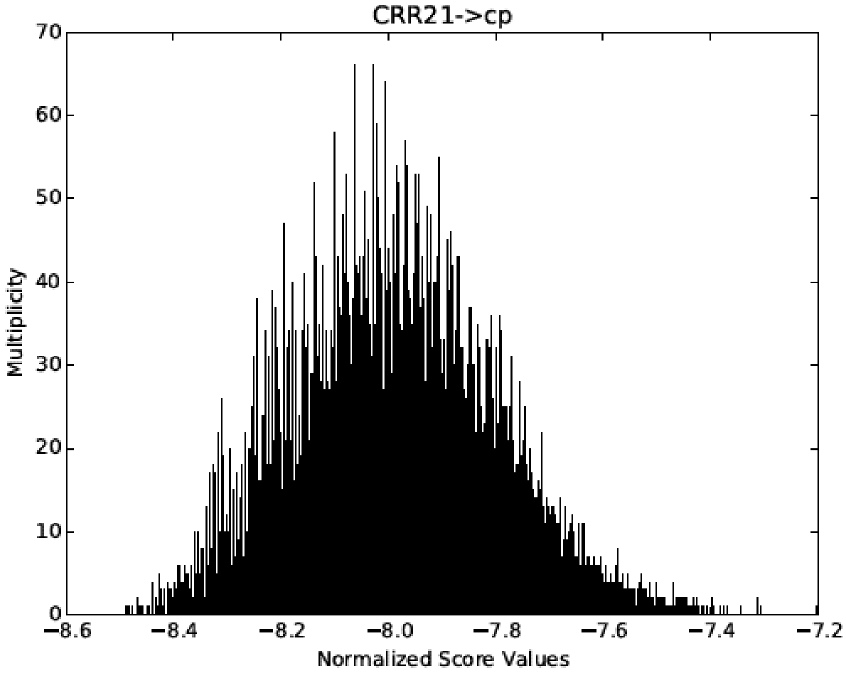

CRR21->cp
Multiplicity
Normalized Score Values
70
60
50
40
30
20
10
0
−8.6
−8.4
−8.2
−8.0
−7.8
−7.6
−7.4
−7.2

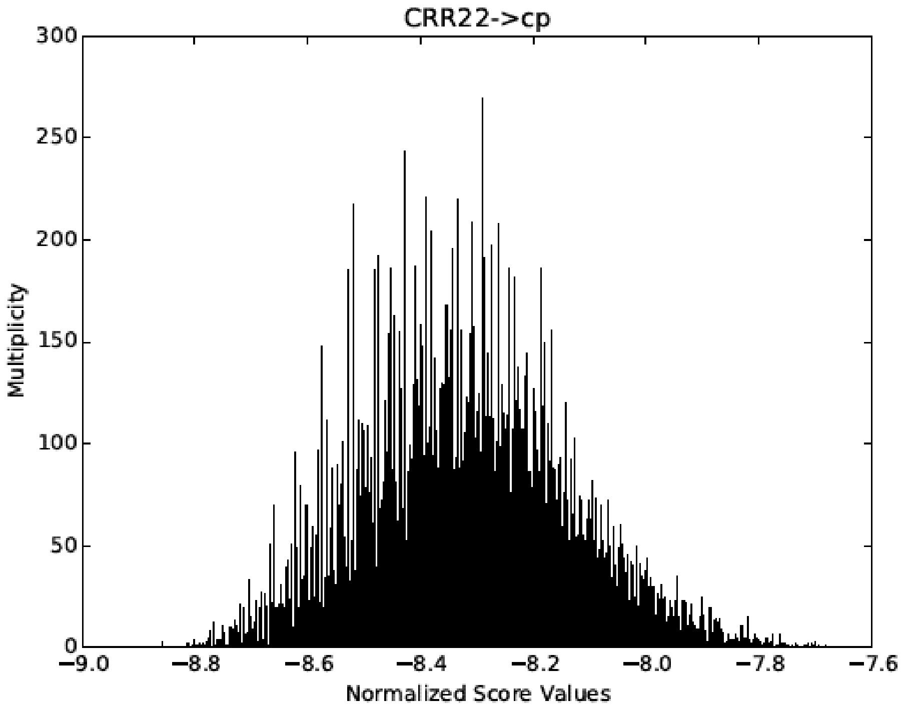
CRR22->cp
Multiplicity
Normalized Score Values
300
250
200
150
100
50
0
−9.0
−8.8
−8.6
−8.4
−8.2
−8.0
−7.8
−7.6

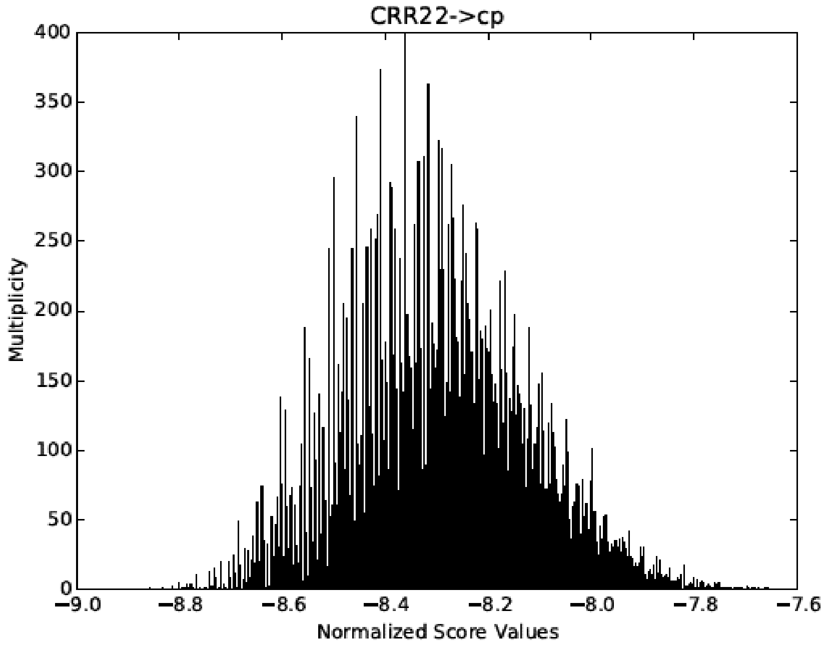
CRR22->cp
Multiplicity
Normalized Score Values
400
350
300
250
200
150
100
50
0
−9.0
−8.8
−8.6
−8.4
−8.2
−8.0
−7.8
−7.6

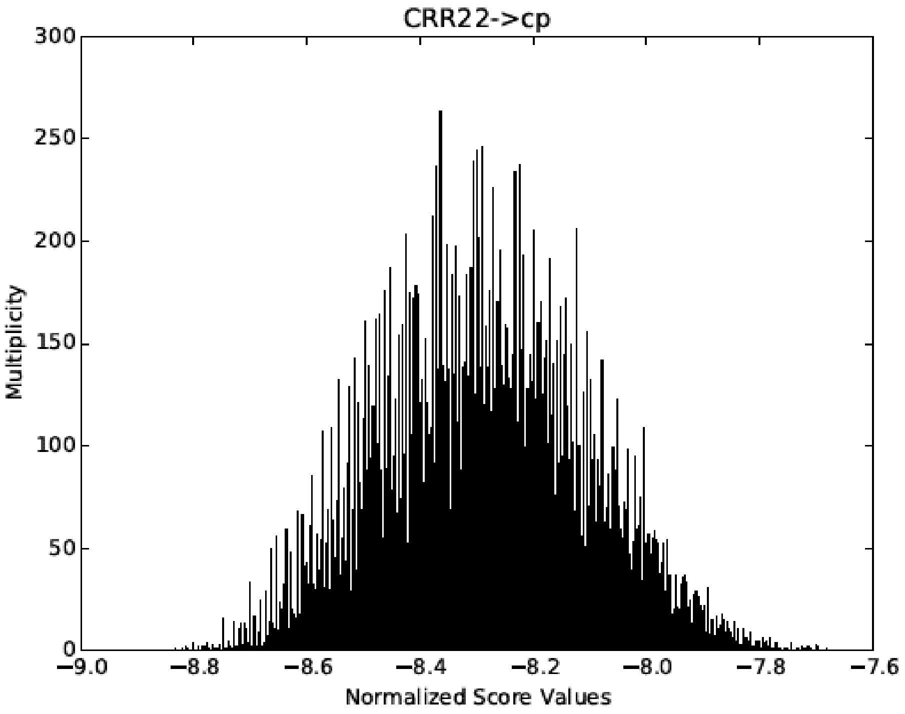

CRR22->cp
Multiplicity
Normalized Score Values
300
250
200
150
100
50
0
−9.0
−8.8
−8.6
−8.4
−8.2
−8.0
−7.8
−7.6

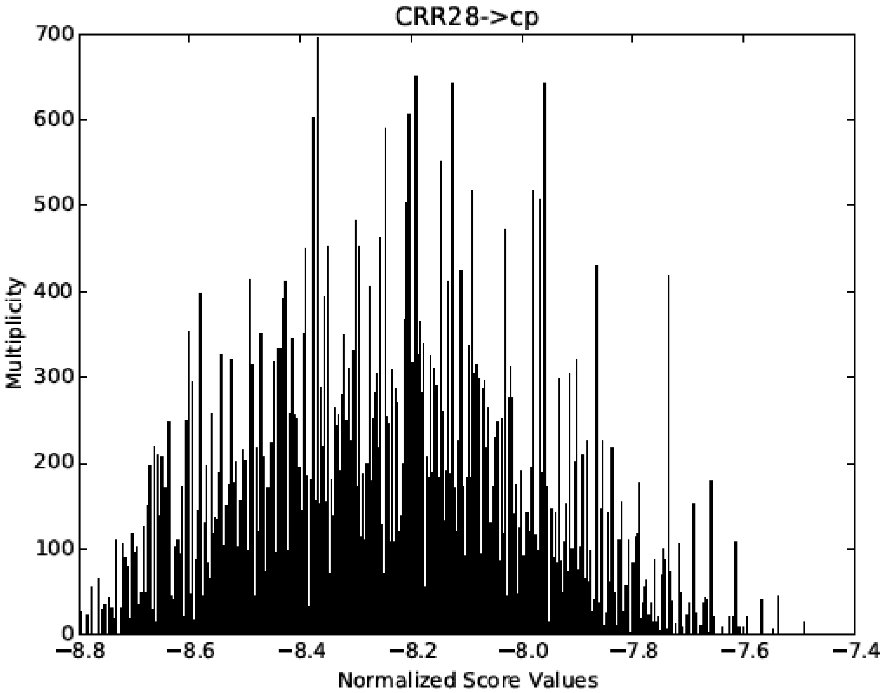

CRR28->cp
Multiplicity
Normalized Score Values
700
600
500
400
300
200
100
0
−8.8
−8.6
−8.4
−8.2
−8.0
−7.8
−7.6
−7.4

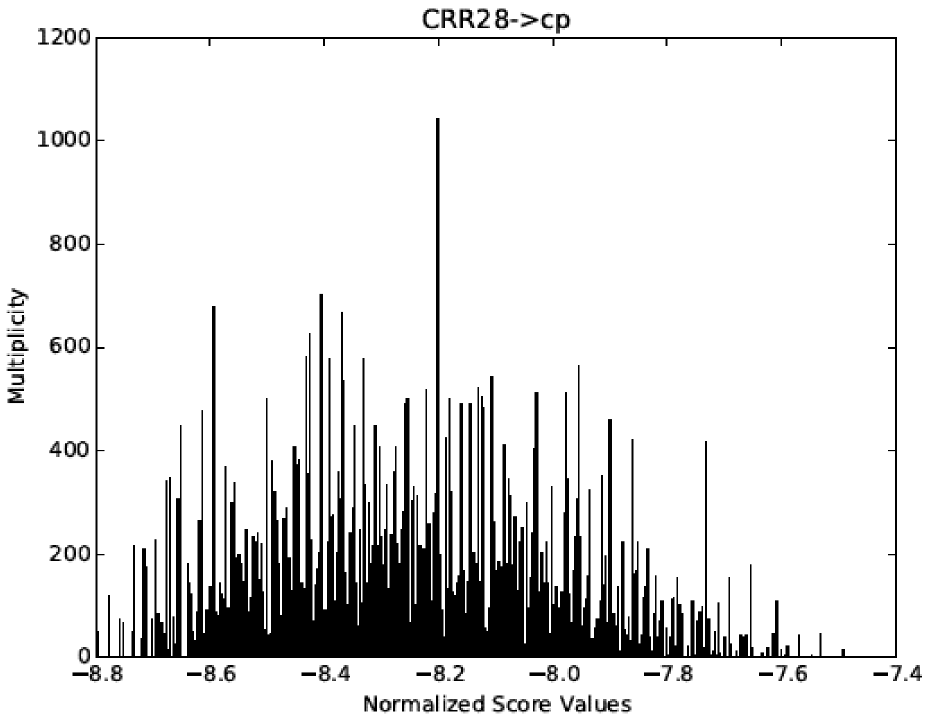
CRR28->cp
Multiplicity
Normalized Score Values
1200
1000
800
600
400
200
0
−8.8
−8.6
−8.4
−8.2
−8.0
−7.8
−7.6
−7.4

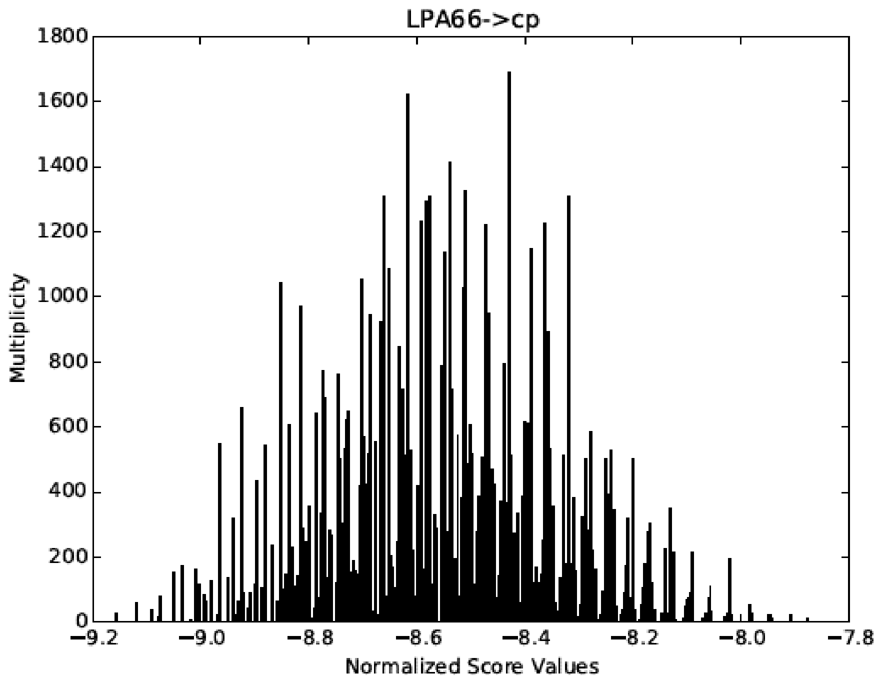
LPA66->cp
Multiplicity
Normalized Score Values
1800
1600
1400
1200
1000
800
600
400
200
0
−9.2
−9.0
−8.8
−8.6
−8.4
−8.2
−8.0
−7.8

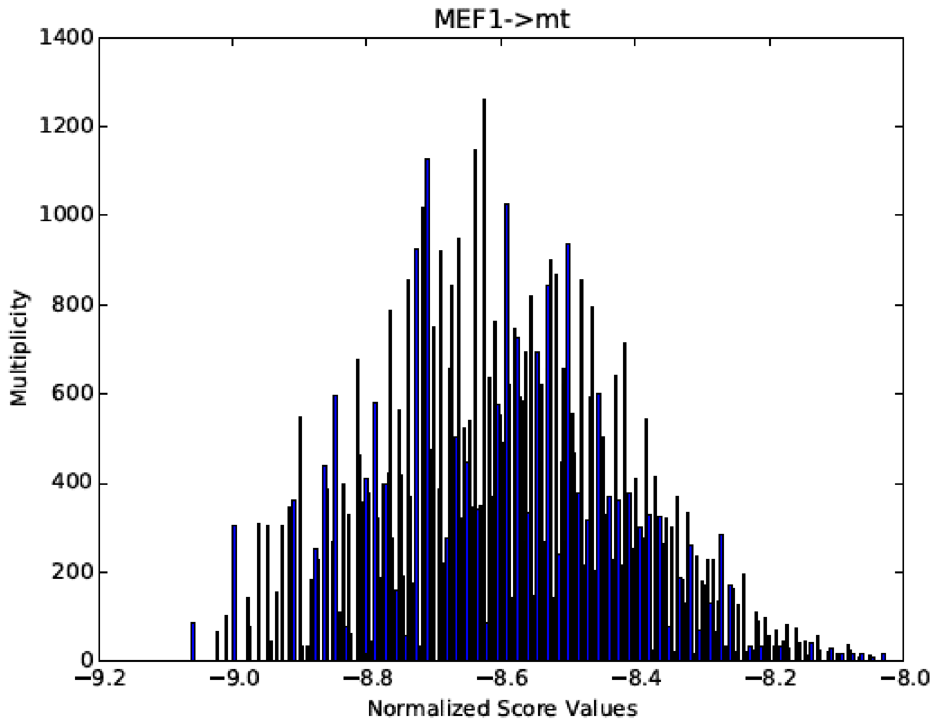
MEF1->mt
Multiplicity
Normalized Score Values
1400
1200
1000
800
600
400
200
0
−9.2
−9.0
−8.8
−8.6
−8.4
−8.2
−8.0

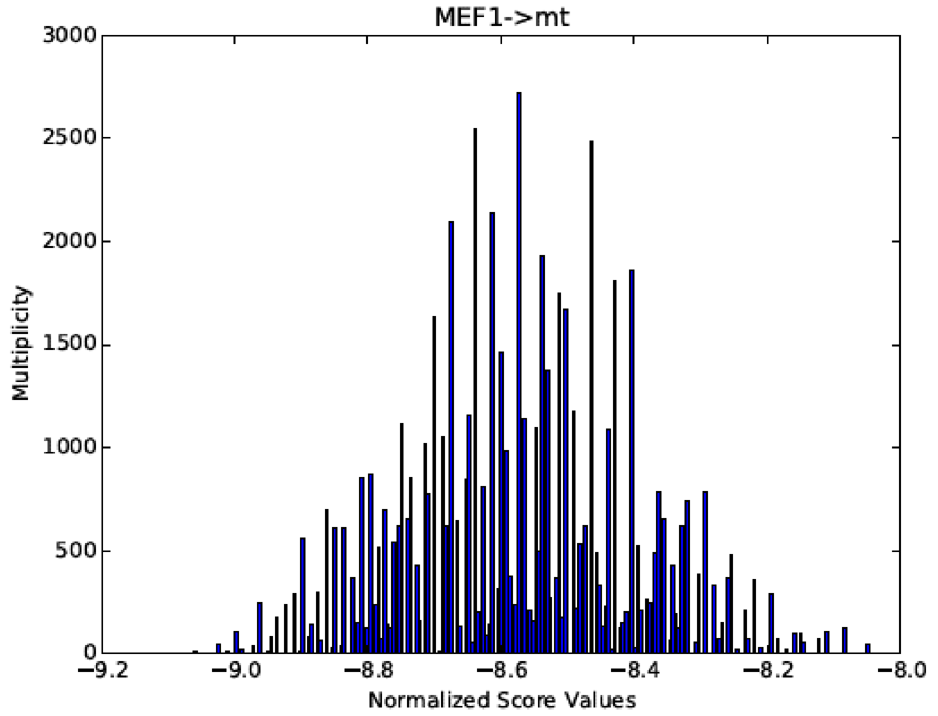
MEF1->mt
Multiplicity
Normalized Score Values
3000
2500
2000
1500
1000
500
0
−9.2
−9.0
−8.8
−8.6
−8.4
−8.2
−8.0

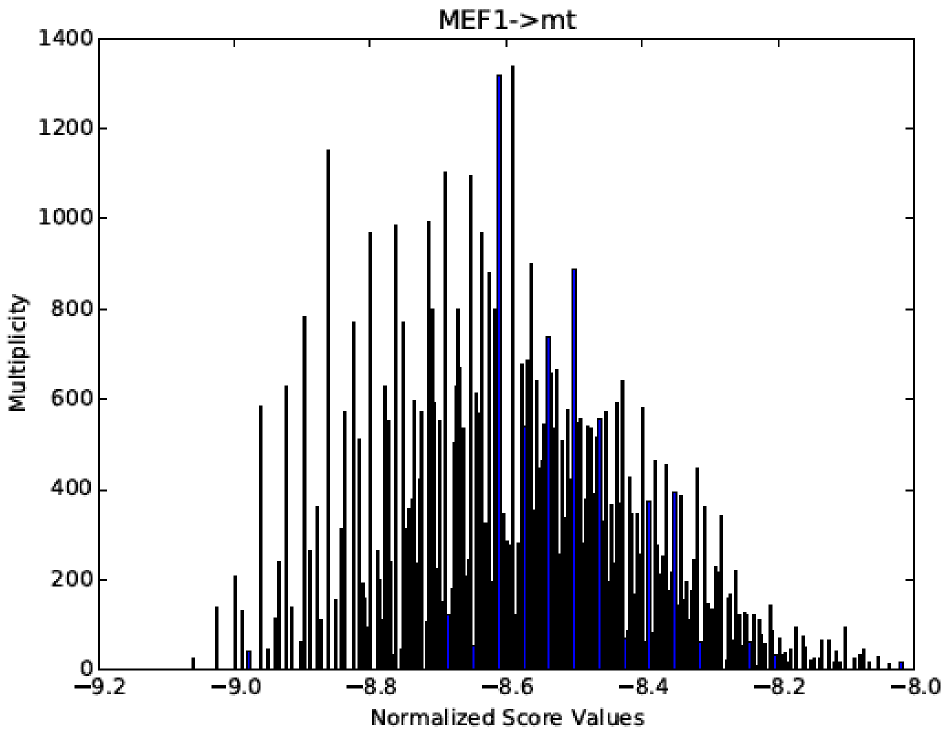
MEF1->mt
Multiplicity
Normalized Score Values
1400
1200
1000
800
600
400
200
0
−9.2
−9.0
−8.8
−8.6
−8.4
−8.2
−8.0

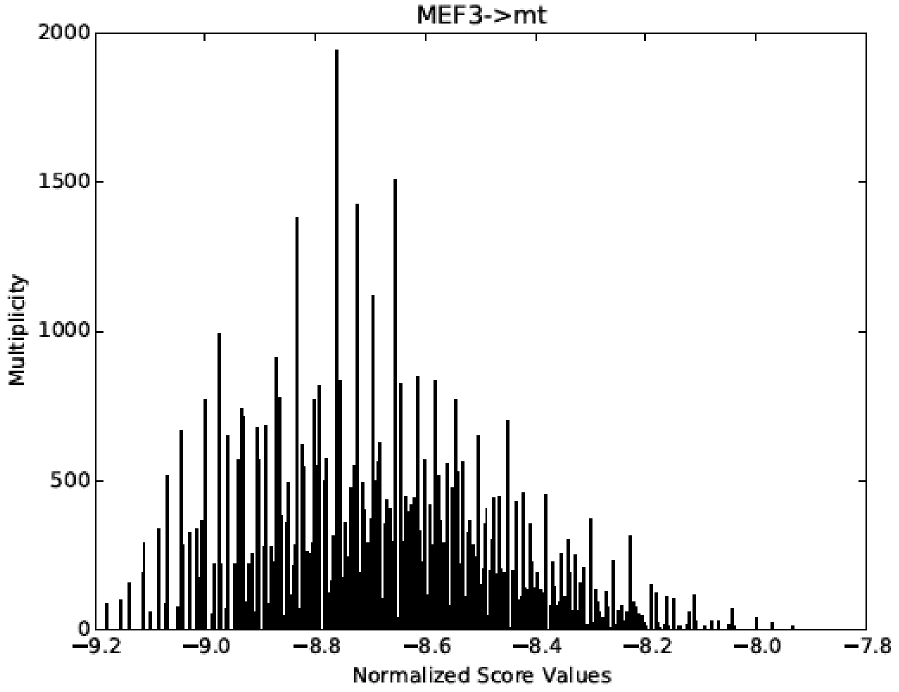
MEF3->mt
Multiplicity
Normalized Score Values
2000
1500
1000
500
0
−9.2
−9.0
−8.8
−8.6
−8.4
−8.2
−8.0
−7.8

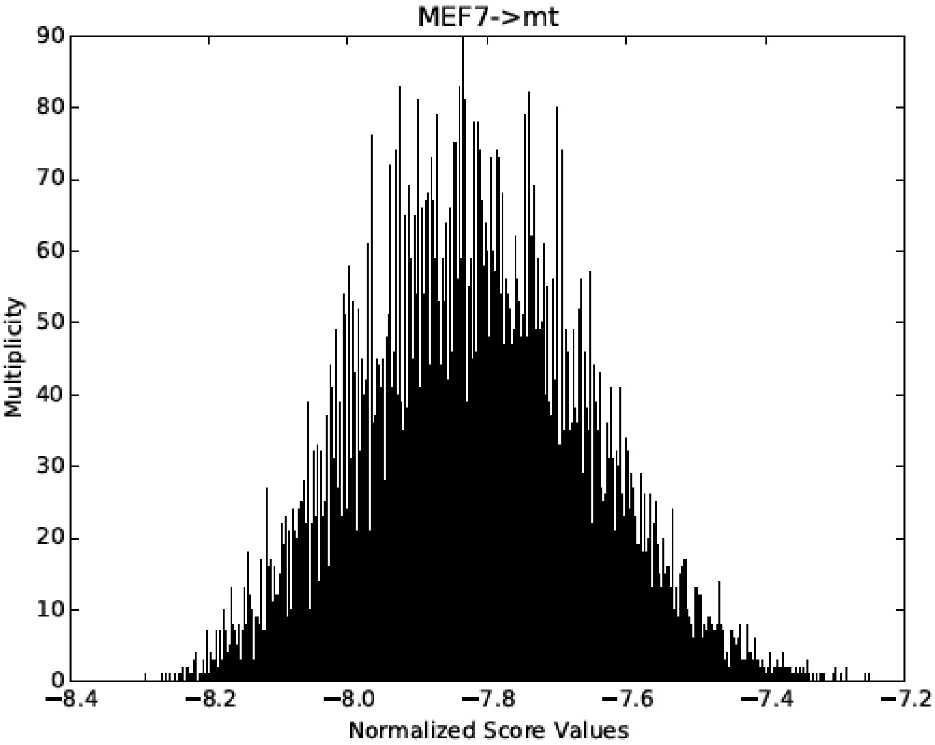
MEF7->mt
Multiplicity
Normalized Score Values
90
80
70
60
50
40
30
20
10
0
−8.4
−8.2
−8.0
−7.8
−7.6
−7.4
−7.2

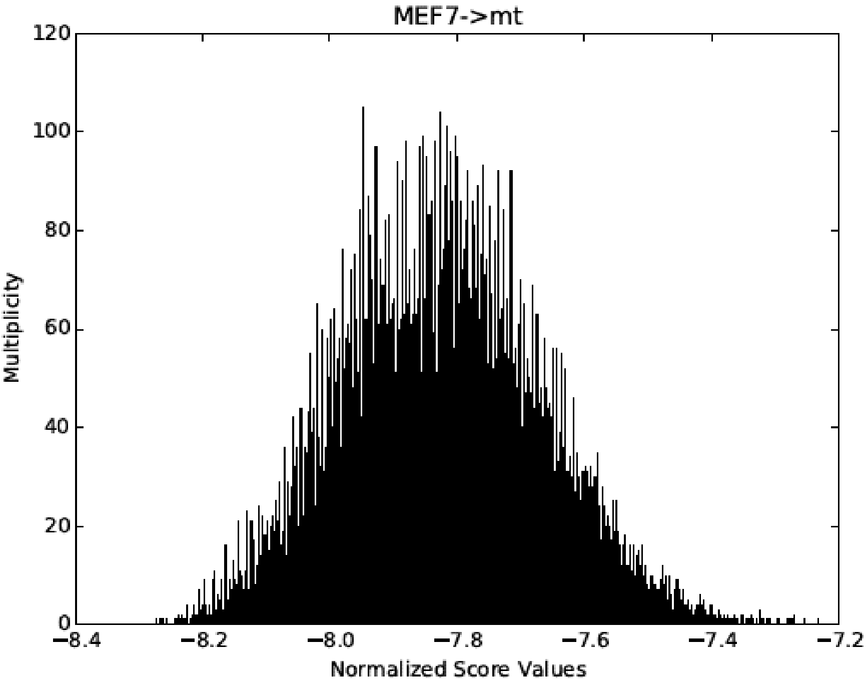
MEF7->mt
Multiplicity
Normalized Score Values
120
100
80
60
40
20
0
−8.4
−8.2
−8.0
−7.8
−7.6
−7.4
−7.2

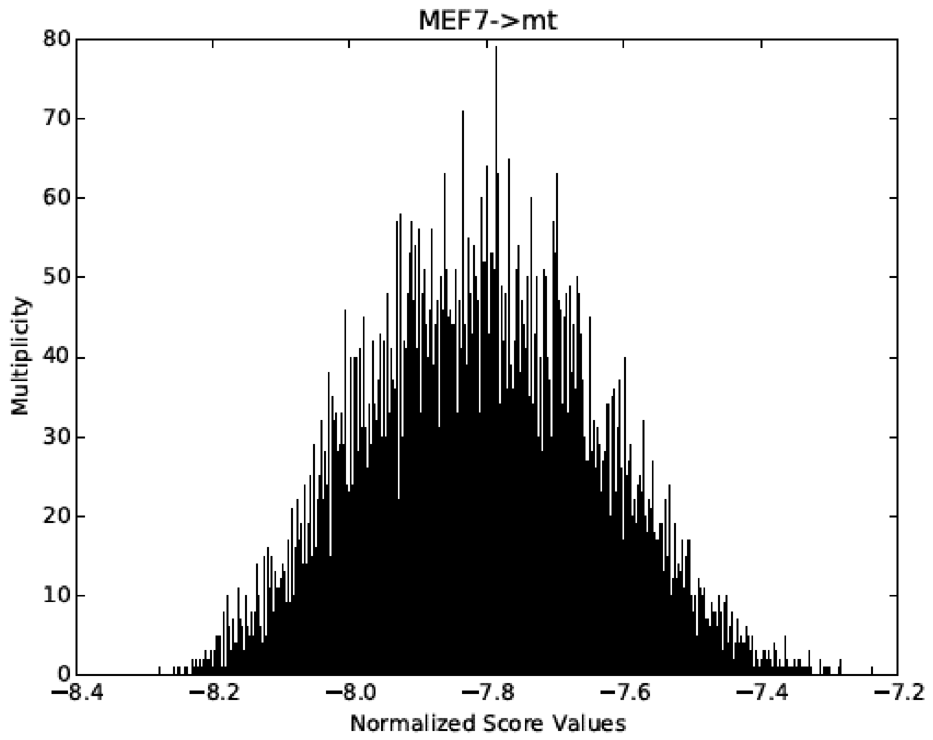
MEF7->mt
Multiplicity
80
70
60
50
40
30
20
10
0
−8.4
−8.2
−8.0
−7.8
−7.6
−7.4
−7.2
Normalized Score Values

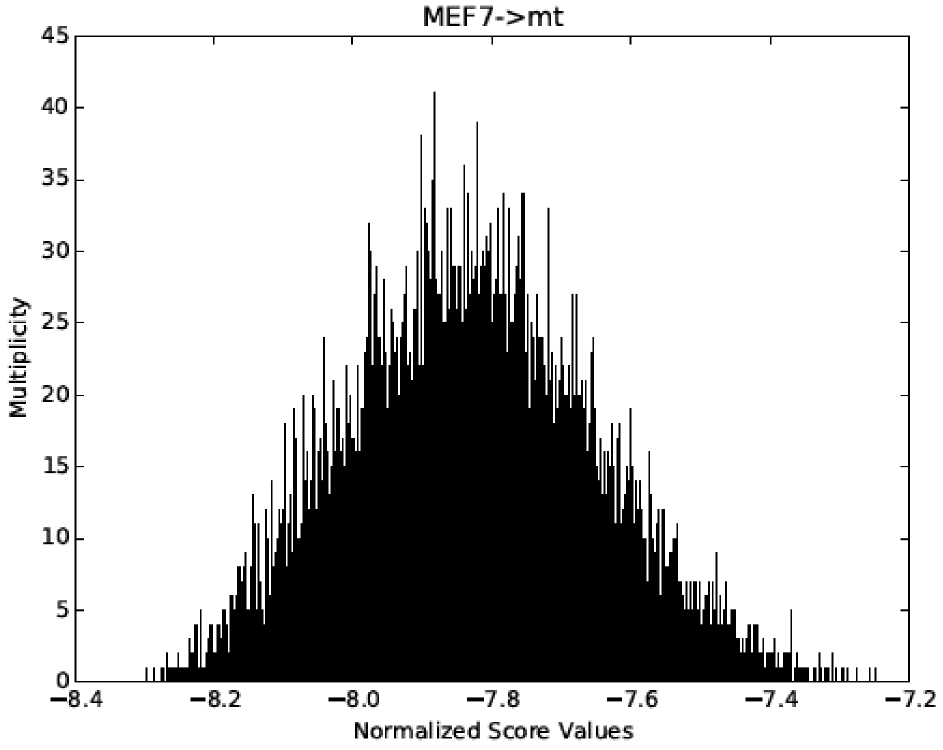
MEF7->mt
Multiplicity
45
40
35
30
25
20
15
10
5
0
−8.4
−8.2
−8.0
−7.8
−7.6
−7.4
−7.2
Normalized Score Values

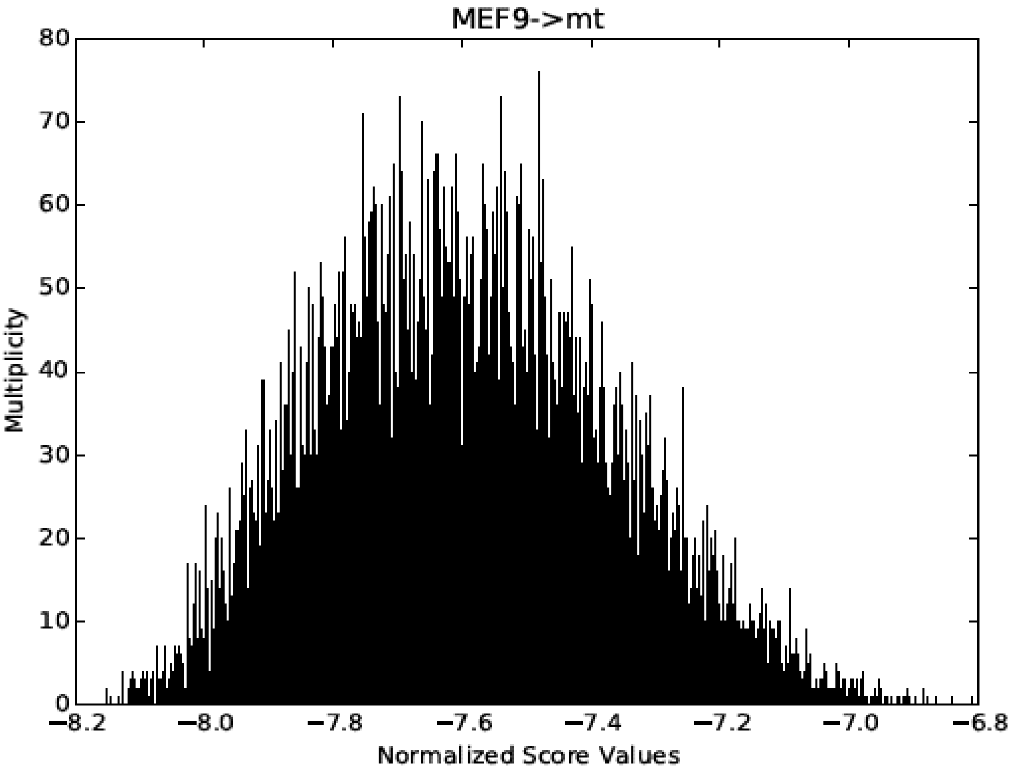
MEF9->mt
Multiplicity
Normalized Score Values
0
10
20
30
40
50
60
70
80
−8.2
−8.0
−7.8
−7.6
−7.4
−7.2
−7.0
−6.8

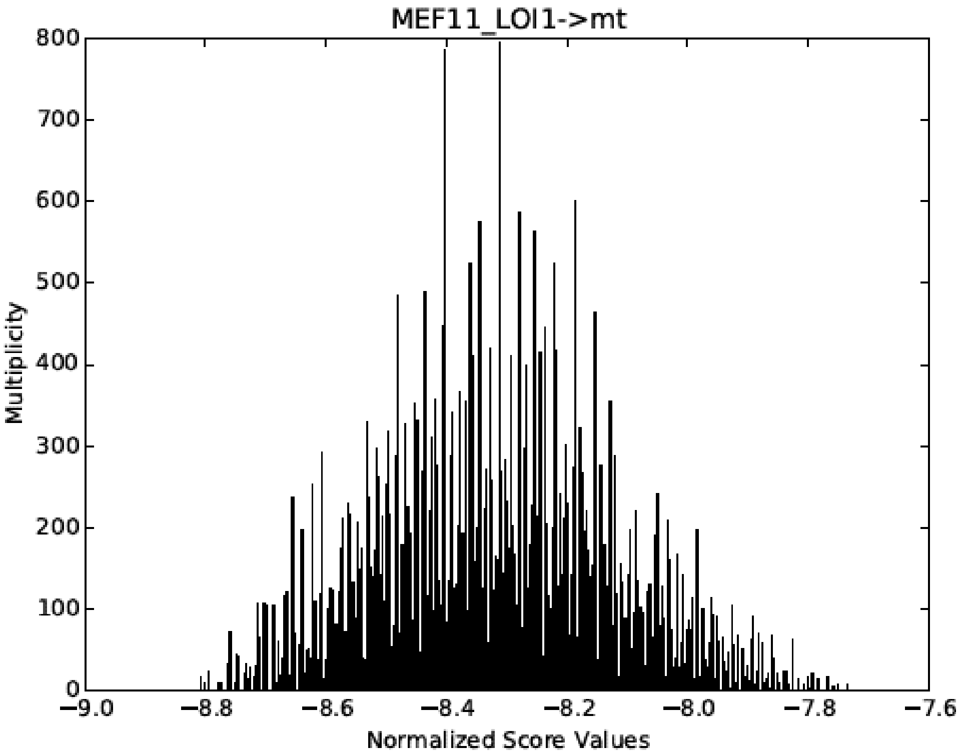
MEF11_LOI1->mt
Multiplicity
Normalized Score Values
0
100
200
300
400
500
600
700
800
−9.0
−8.8
−8.6
−8.4
−8.2
−8.0
−7.8
−7.6

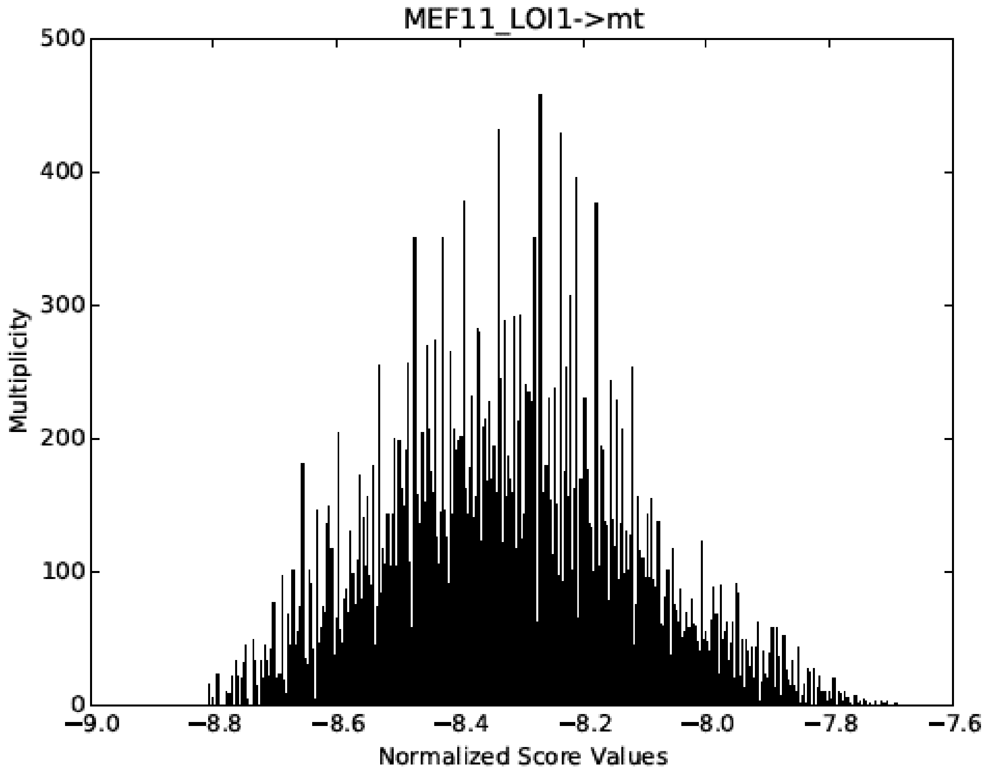
MEF11_LOI1->mt
Multiplicity
Normalized Score Values
500
400
300
200
100
0
−9.0
−8.8
−8.6
−8.4
−8.2
−8.0
−7.8
−7.6

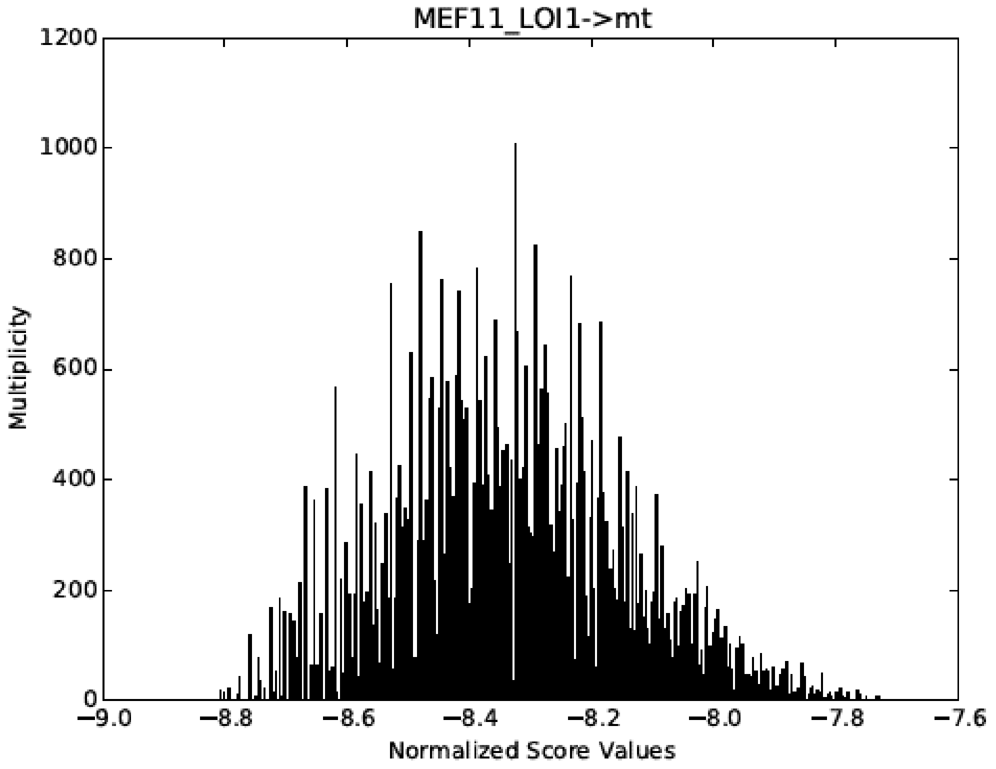
MEF11_LOI1->mt
Multiplicity
Normalized Score Values
1200
1000
800
600
400
200
0
−9.0
−8.8
−8.6
−8.4
−8.2
−8.0
−7.8
−7.6

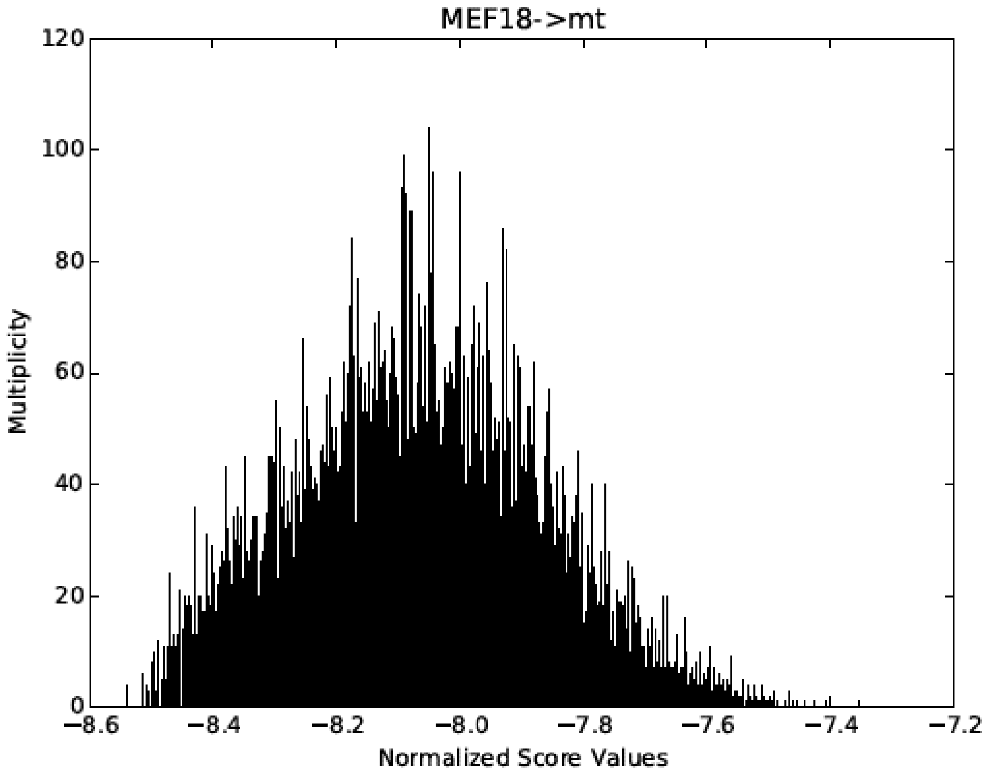
MEF18->mt
120
100
80
60
40
20
0
Multiplicity
−8.6
−8.4
−8.2
−8.0
−7.8
−7.6
−7.4
−7.2
Normalized Score Values

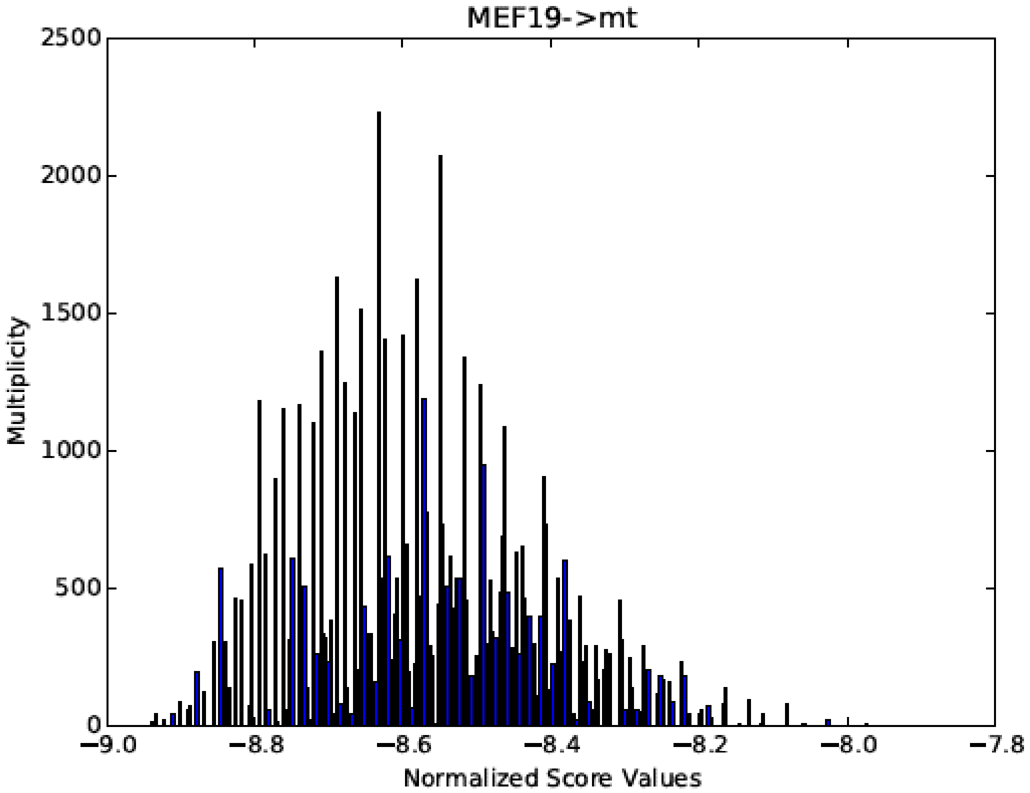
MEF19->mt
2500
2000
1500
1000
500
0
Multiplicity
−9.0
−8.8
−8.6
−8.4
−8.2
−8.0
−7.8
Normalized Score Values

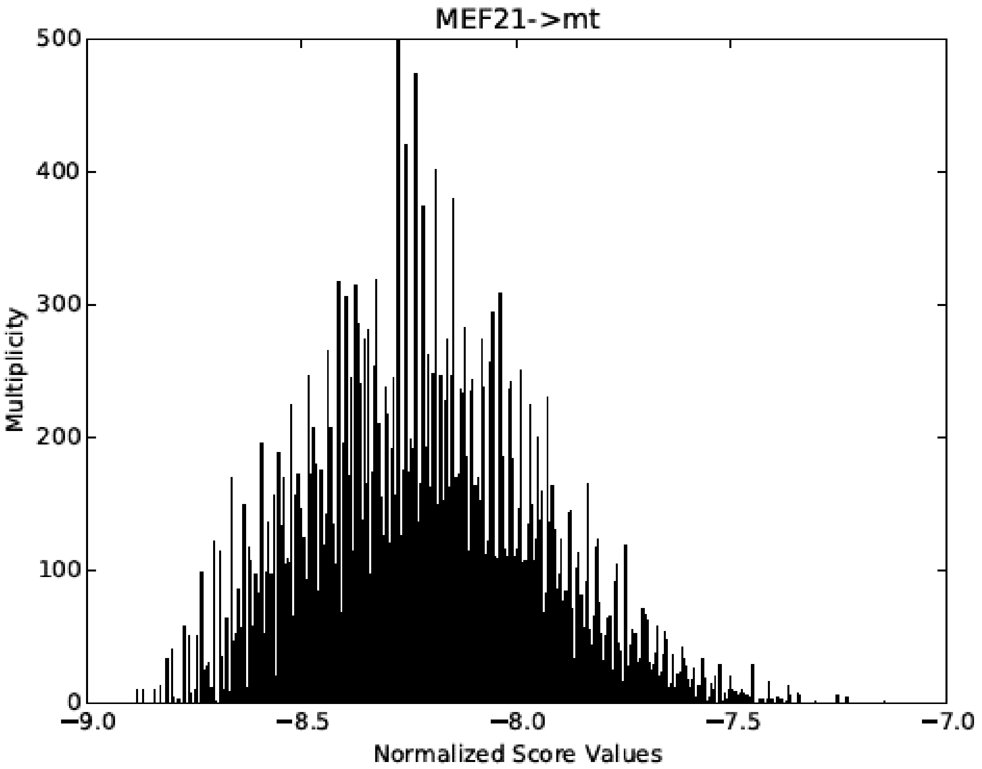
MEF21->mt
Multiplicity
0
100
200
300
400
500
−9.0
−8.5
−8.0
−7.5
−7.0
Normalized Score Values

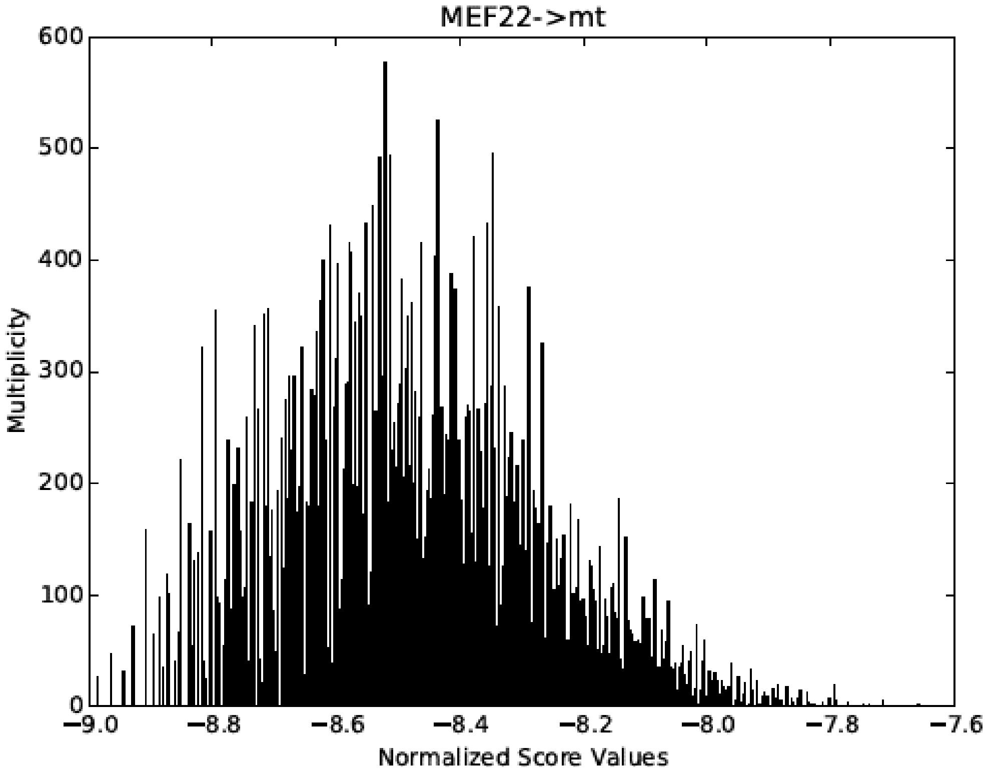
MEF22->mt
Multiplicity
0
100
200
300
400
500
600
−9.0
−8.8
−8.6
−8.4
−8.2
−8.0
−7.8
−7.6
Normalized Score Values

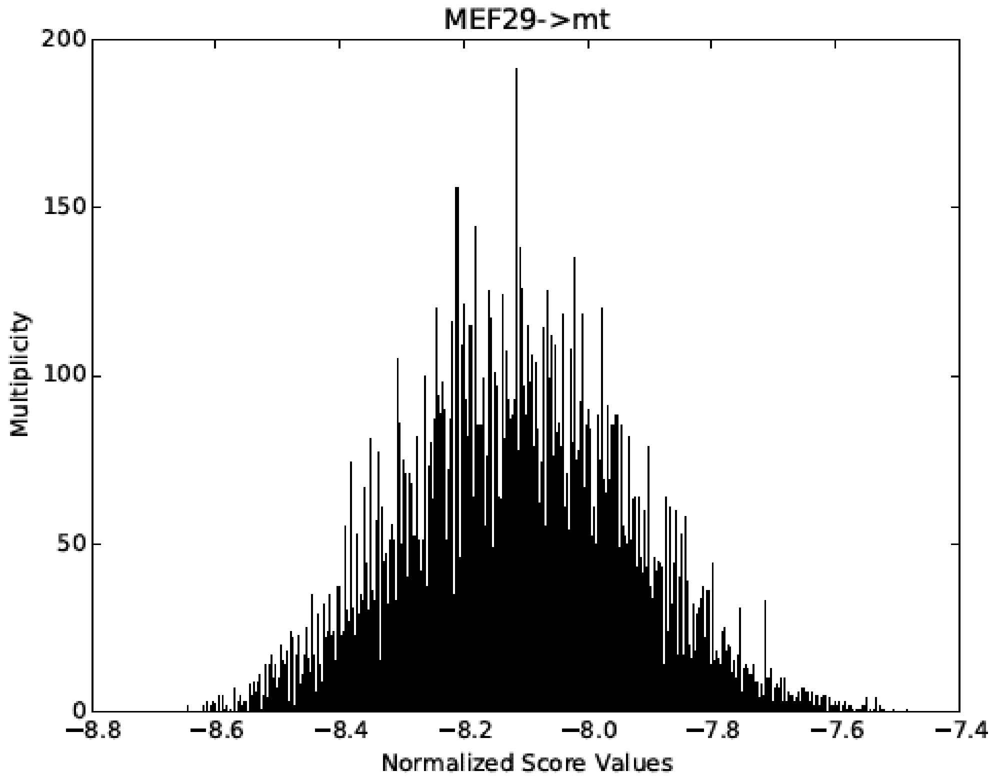
MEF29->mt
Multiplicity
Normalized Score Values
200
150
100
50
0
−8.8
−8.6
−8.4
−8.2
−8.0
−7.8
−7.6
−7.4

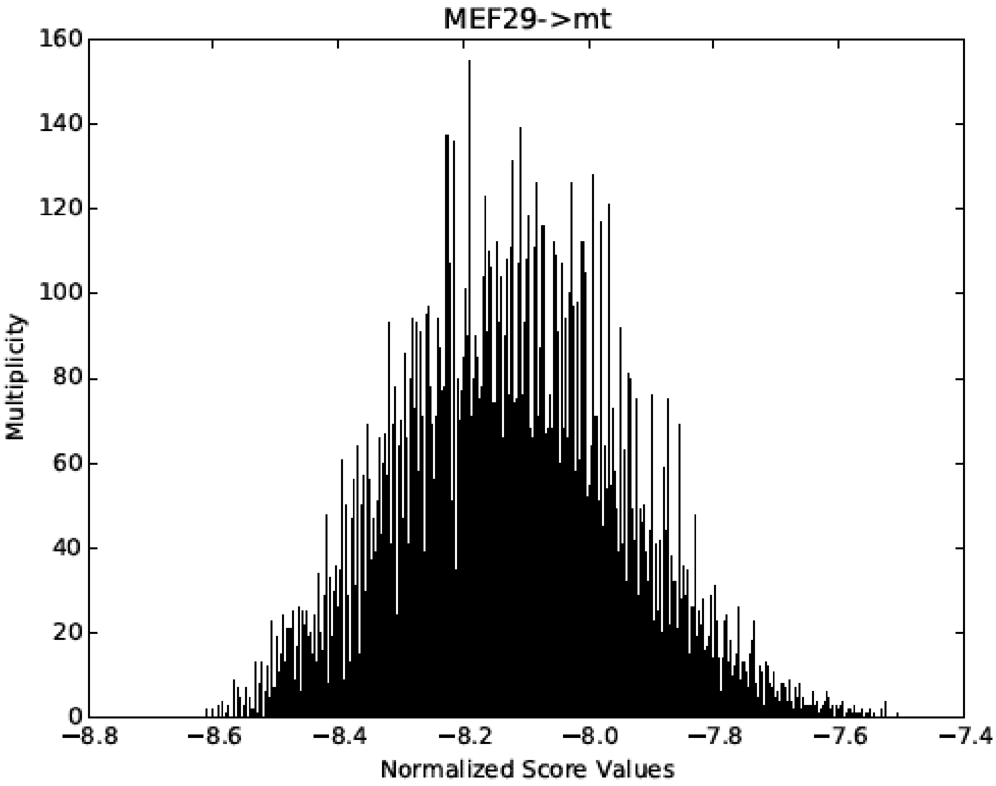
MEF29->mt
Multiplicity
Normalized Score Values
160
140
120
100
80
60
40
20
0
−8.8
−8.6
−8.4
−8.2
−8.0
−7.8
−7.6
−7.4

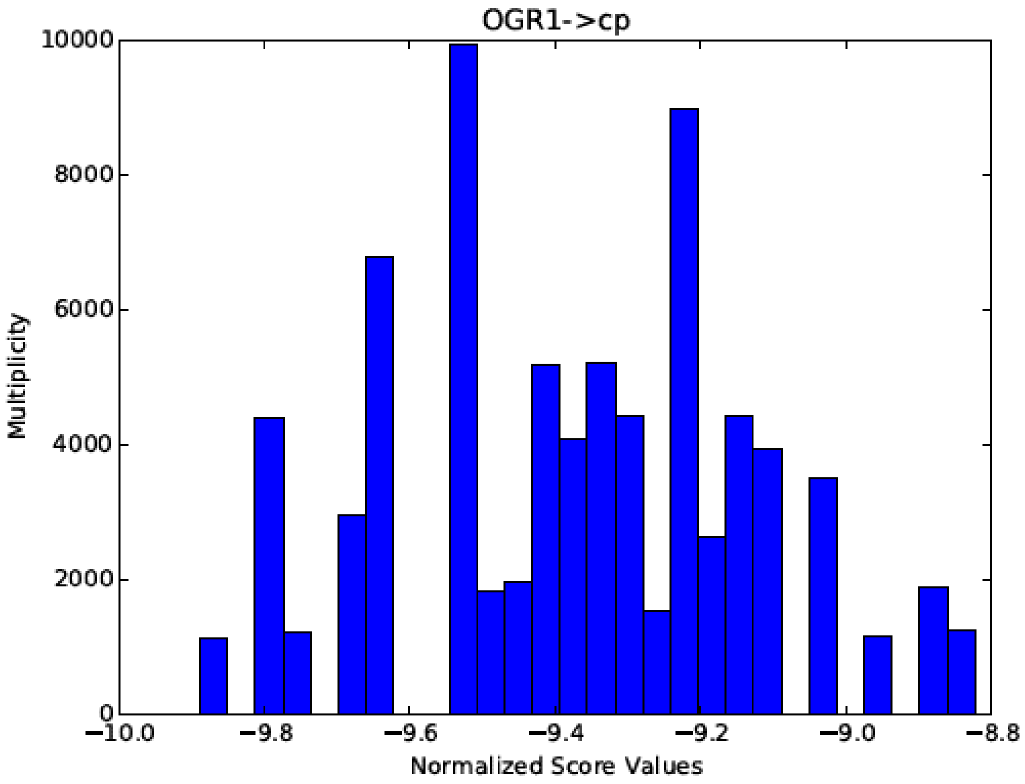
OGR1->cp
Multiplicity
Normalized Score Values
10000
8000
6000
4000
2000
0
−10.0
−9.8
−9.6
−9.4
−9.2
−9.0
−8.8

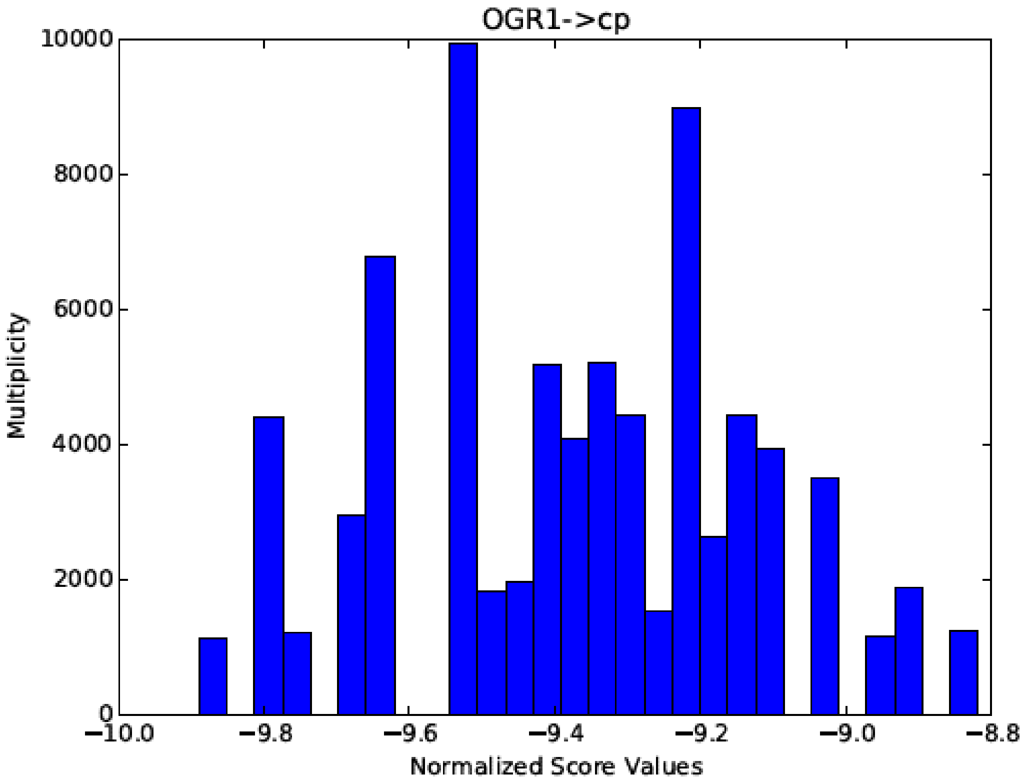
OGR1->cp
Multiplicity
Normalized Score Values
10000
8000
6000
4000
2000
0
−10.0
−9.8
−9.6
−9.4
−9.2
−9.0
−8.8

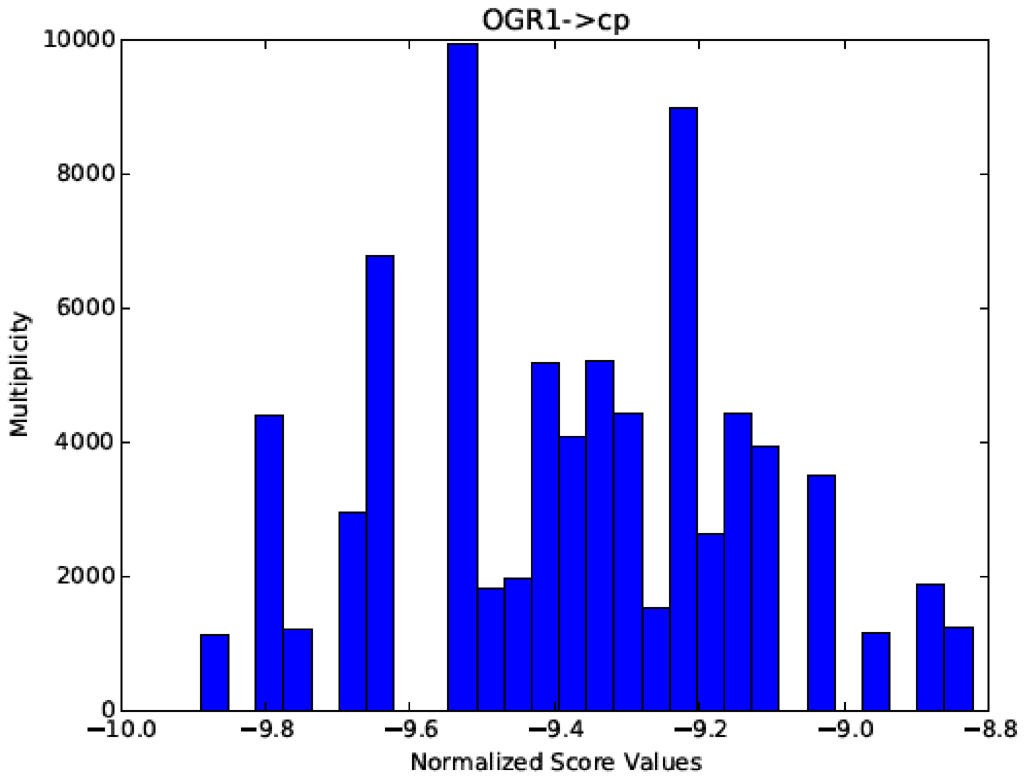
OGR1->cp
Multiplicity
Normalized Score Values
0
2000
4000
6000
8000
10000
−10.0
−9.8
−9.6
−9.4
−9.2
−9.0
−8.8

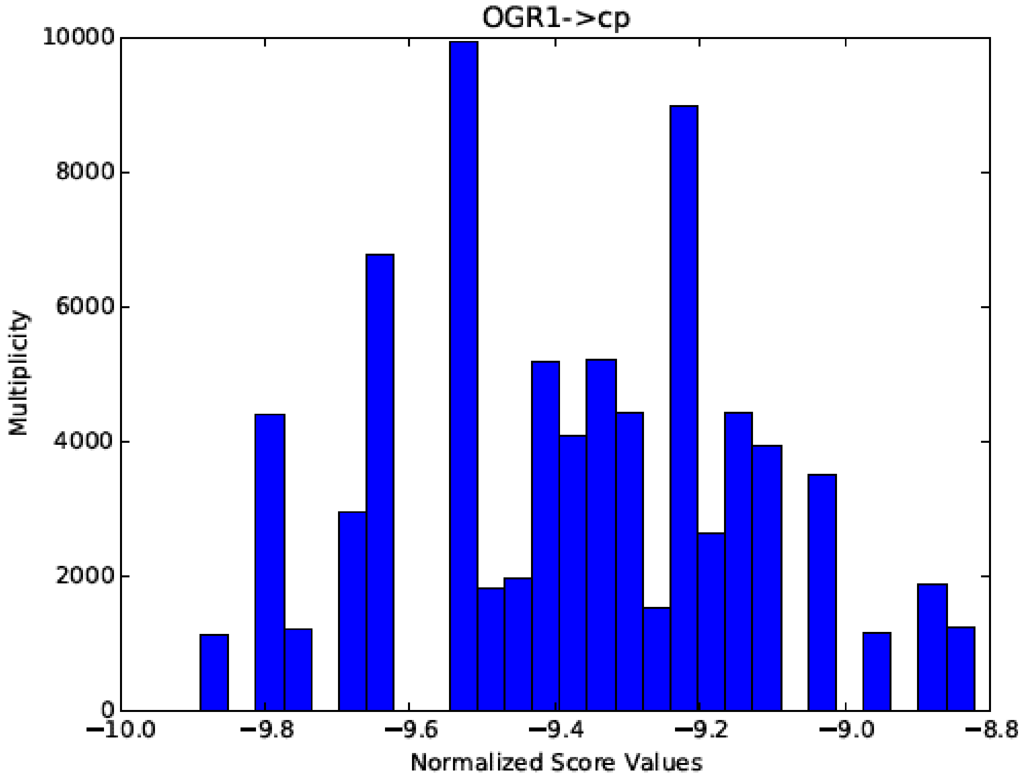
OGR1->cp
Multiplicity
Normalized Score Values
0
2000
4000
6000
8000
10000
−10.0
−9.8
−9.6
−9.4
−9.2
−9.0
−8.8

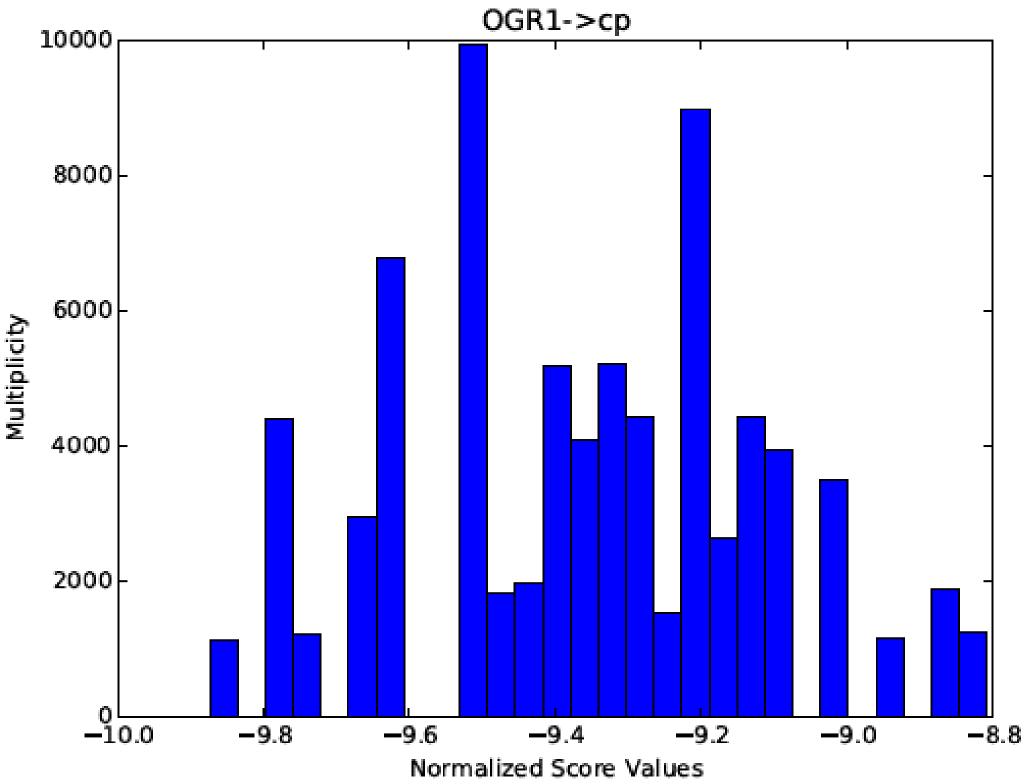
OGR1->cp
Multiplicity
Normalized Score Values
10000
8000
6000
4000
2000
0
−10.0
−9.8
−9.6
−9.4
−9.2
−9.0
−8.8

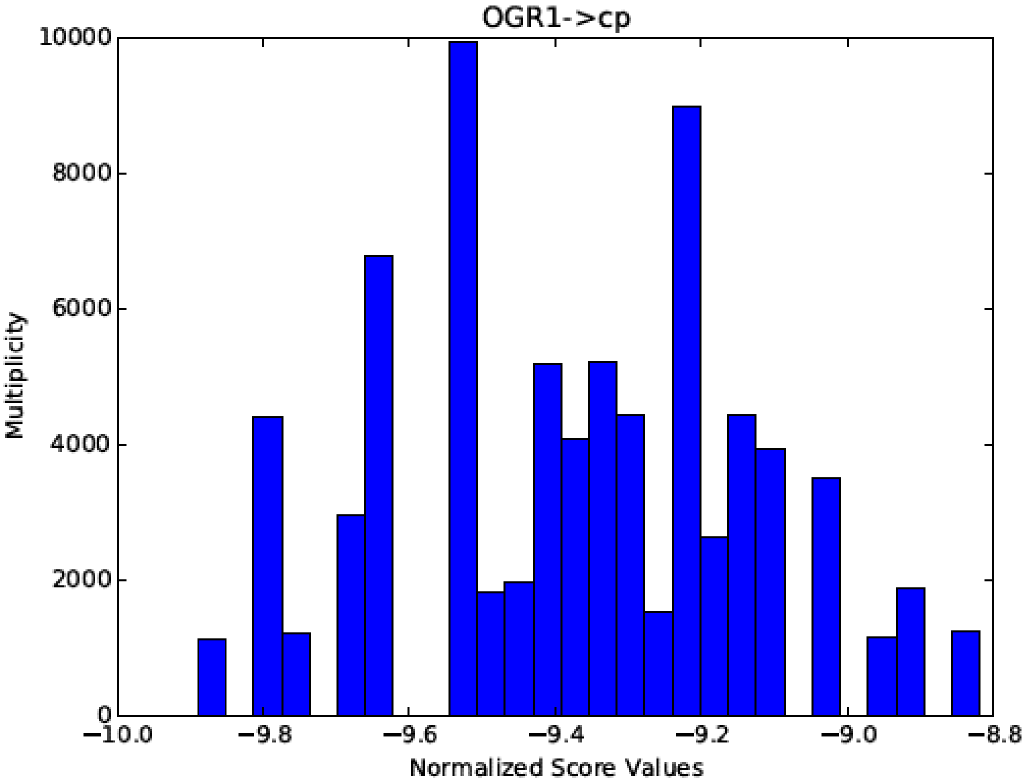
OGR1->cp
Multiplicity
Normalized Score Values
10000
8000
6000
4000
2000
0
−10.0
−9.8
−9.6
−9.4
−9.2
−9.0
−8.8

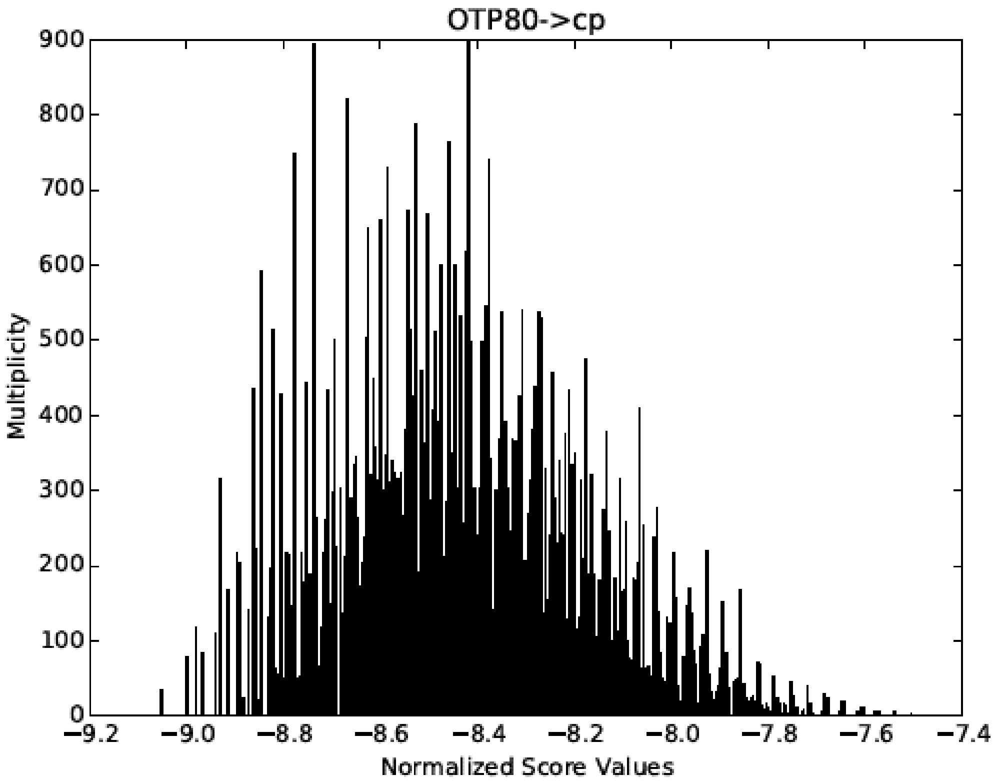
OTP80->cp
900
800
700
600
500
400
300
200
100
0
Multiplicity
−9.2
−9.0
−8.8
−8.6
−8.4
−8.2
−8.0
−7.8
−7.6
−7.4
Normalized Score Values

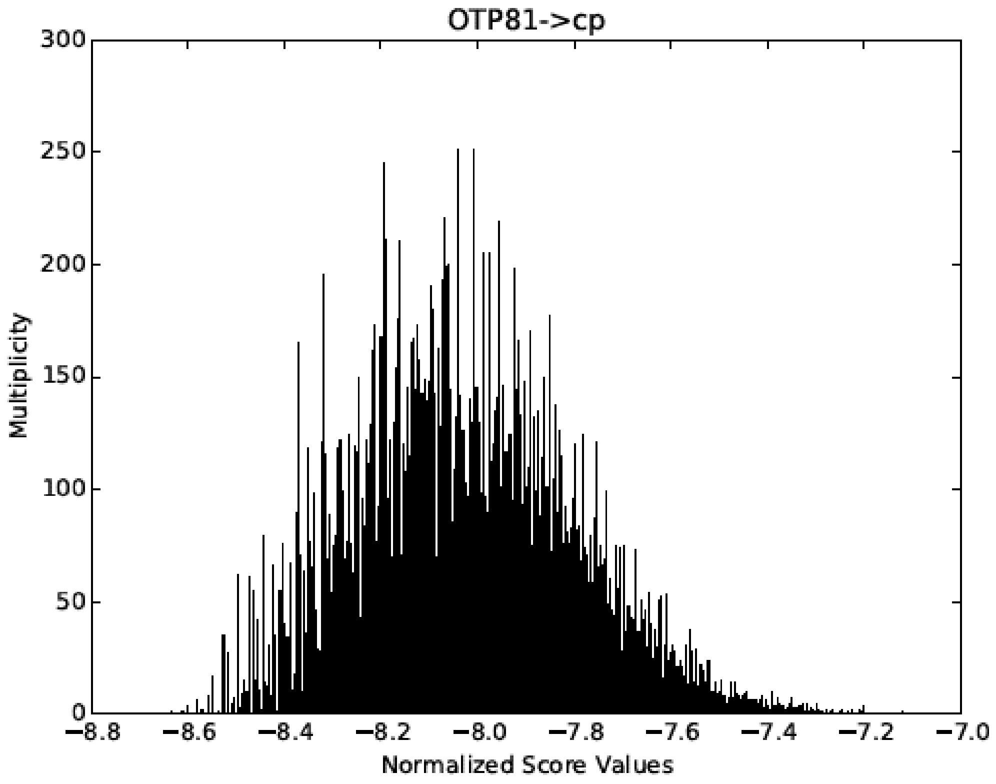
OTP81->cp
300
250
200
150
100
50
0
Multiplicity
−8.8
−8.6
−8.4
−8.2
−8.0
−7.8
−7.6
−7.4
−7.2
−7.0
Normalized Score Values

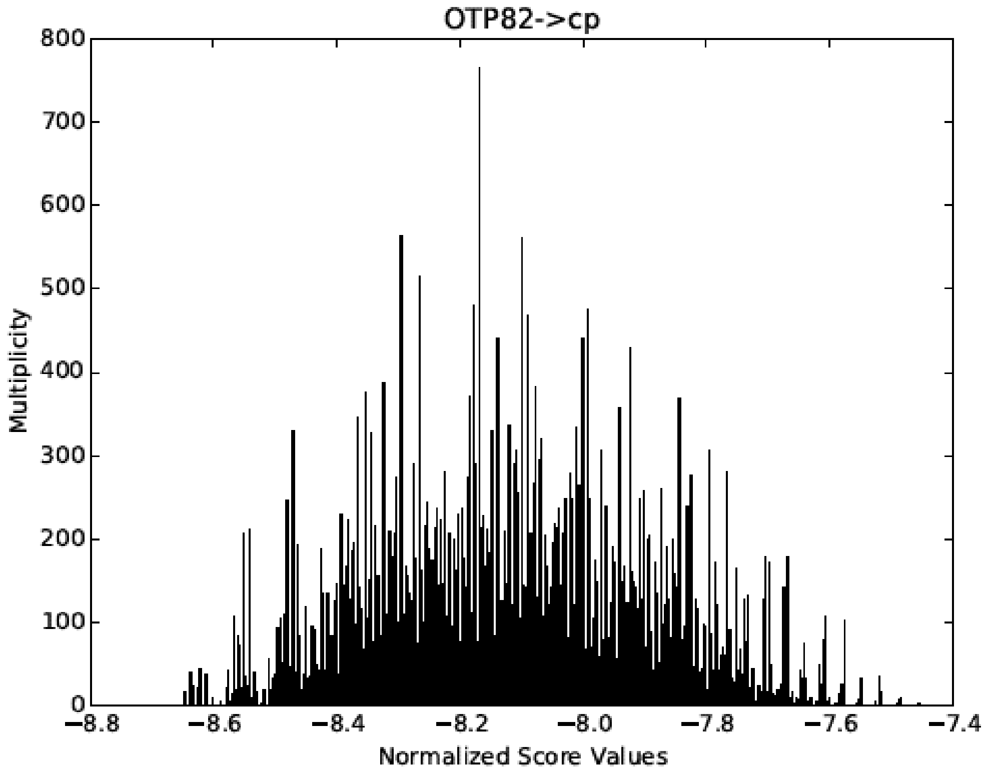
OTP82->cp
Multiplicity
Normalized Score Values
0
100
200
300
400
500
600
700
800
−8.8
−8.6
−8.4
−8.2
−8.0
−7.8
−7.6
−7.4

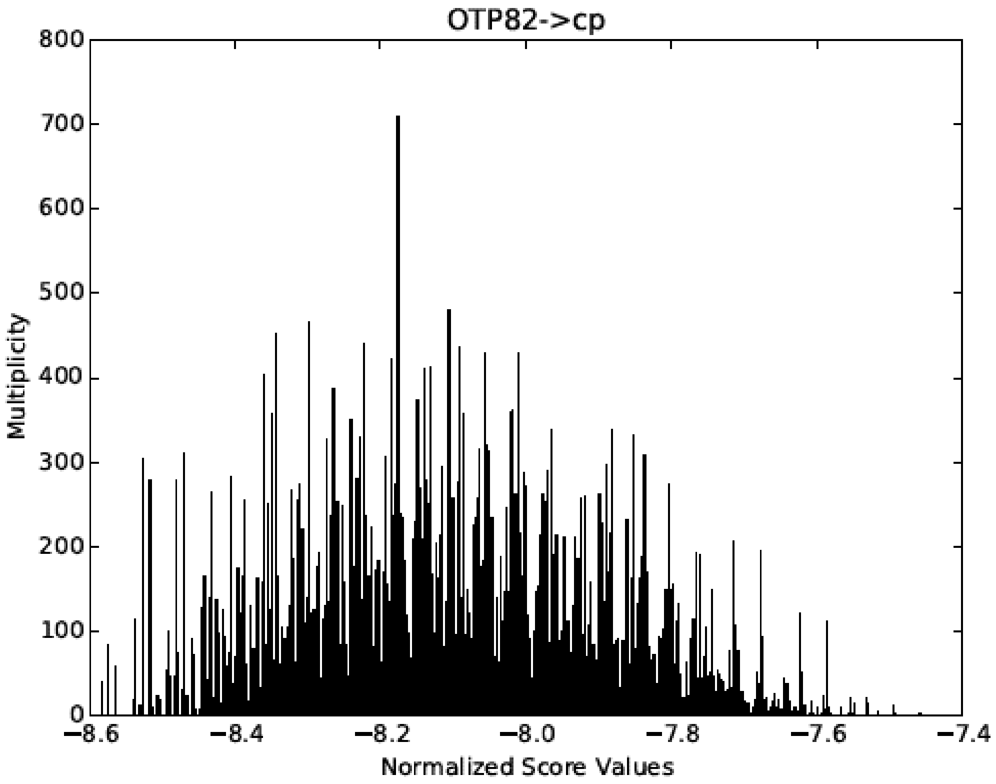
OTP82->cp
Multiplicity
Normalized Score Values
0
100
200
300
400
500
600
700
800
−8.6
−8.4
−8.2
−8.0
−7.8
−7.6
−7.4

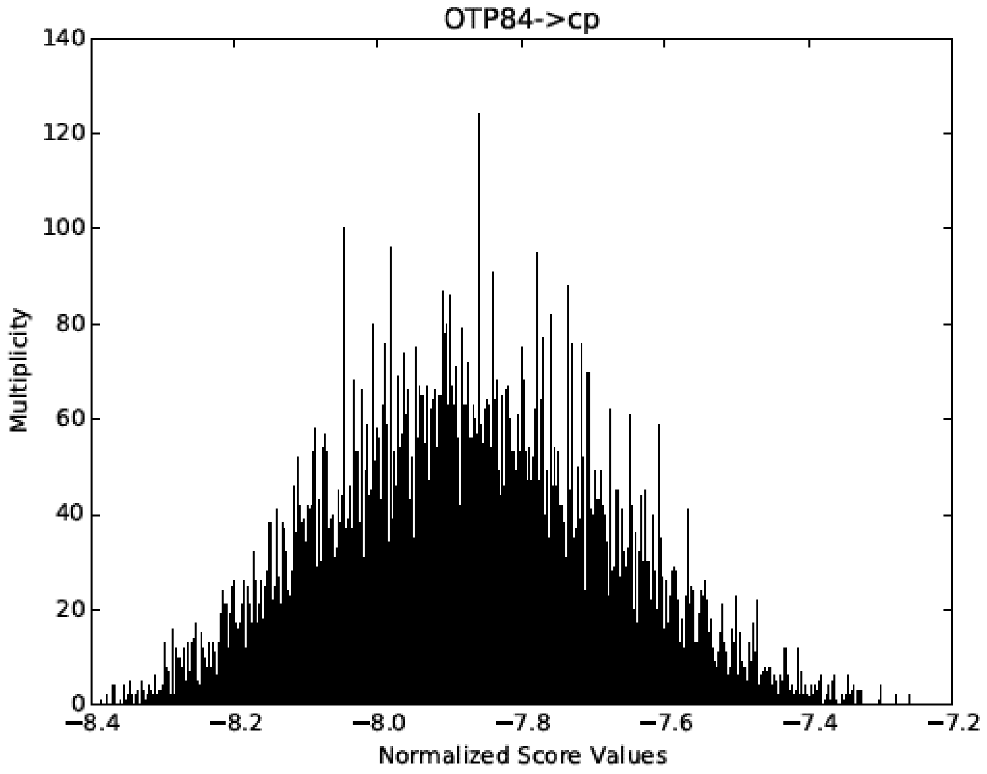
OTP84->cp
Multiplicity
Normalized Score Values
0
20
40
60
80
100
120
140
−8.4
−8.2
−8.0
−7.8
−7.6
−7.4
−7.2

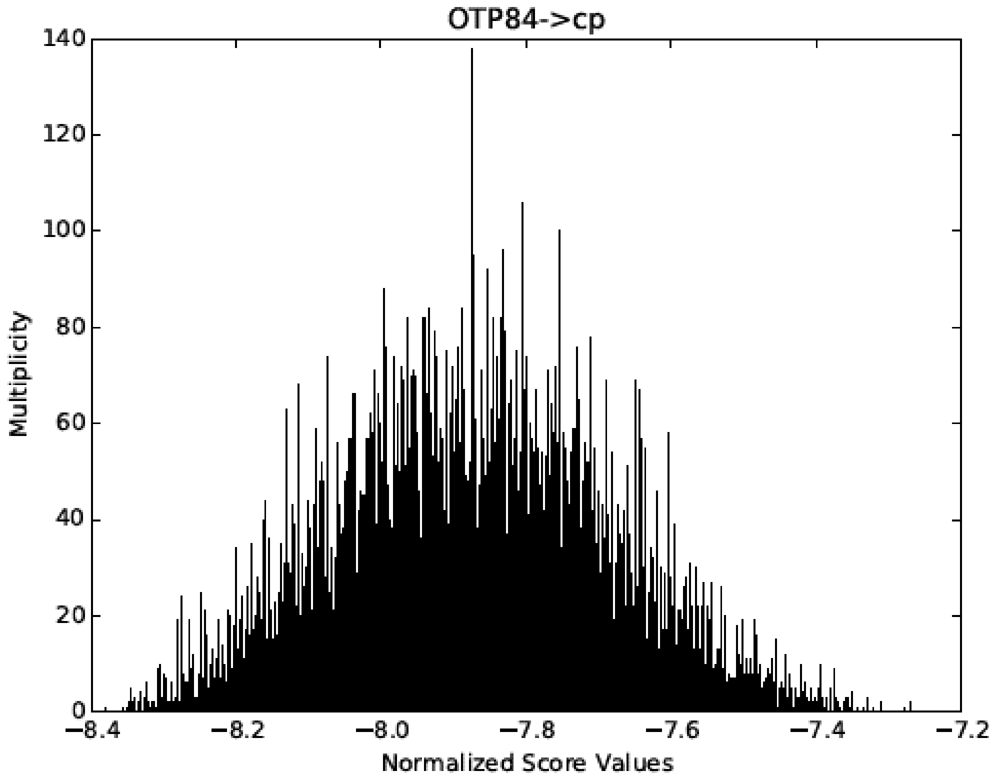
OTP84->cp
Multiplicity
Normalized Score Values
0
20
40
60
80
100
120
140
−8.4
−8.2
−8.0
−7.8
−7.6
−7.4
−7.2

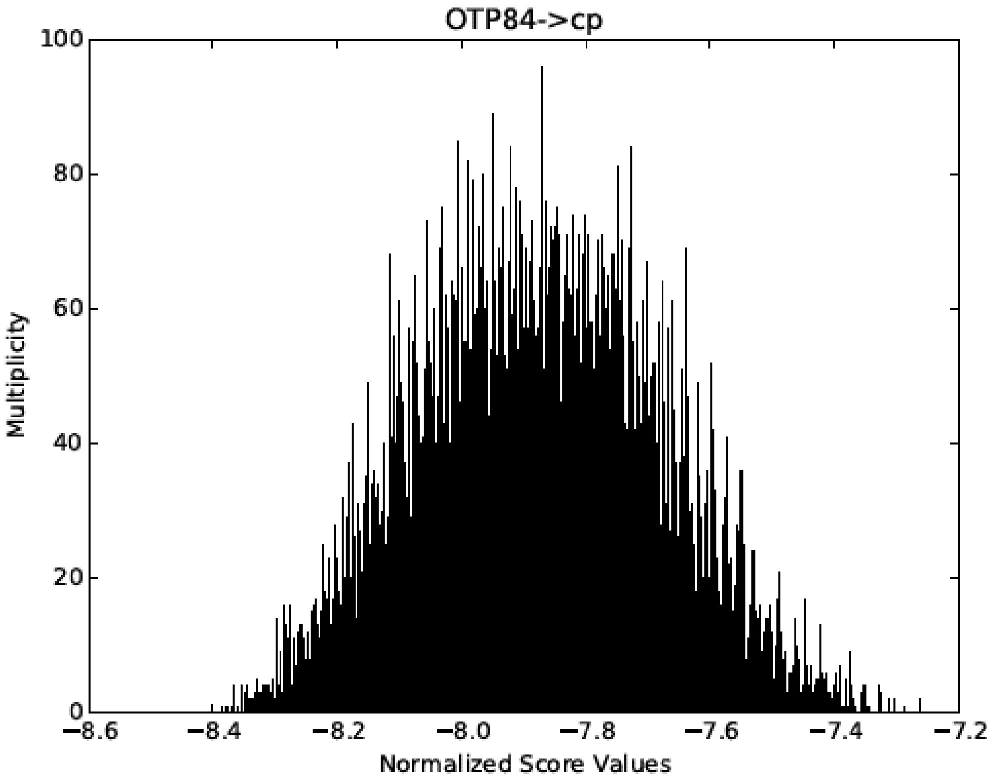
OTP84->cp
Multiplicity
Normalized Score Values
100
80
60
40
20
0
−8.6
−8.4
−8.2
−8.0
−7.8
−7.6
−7.4
−7.2

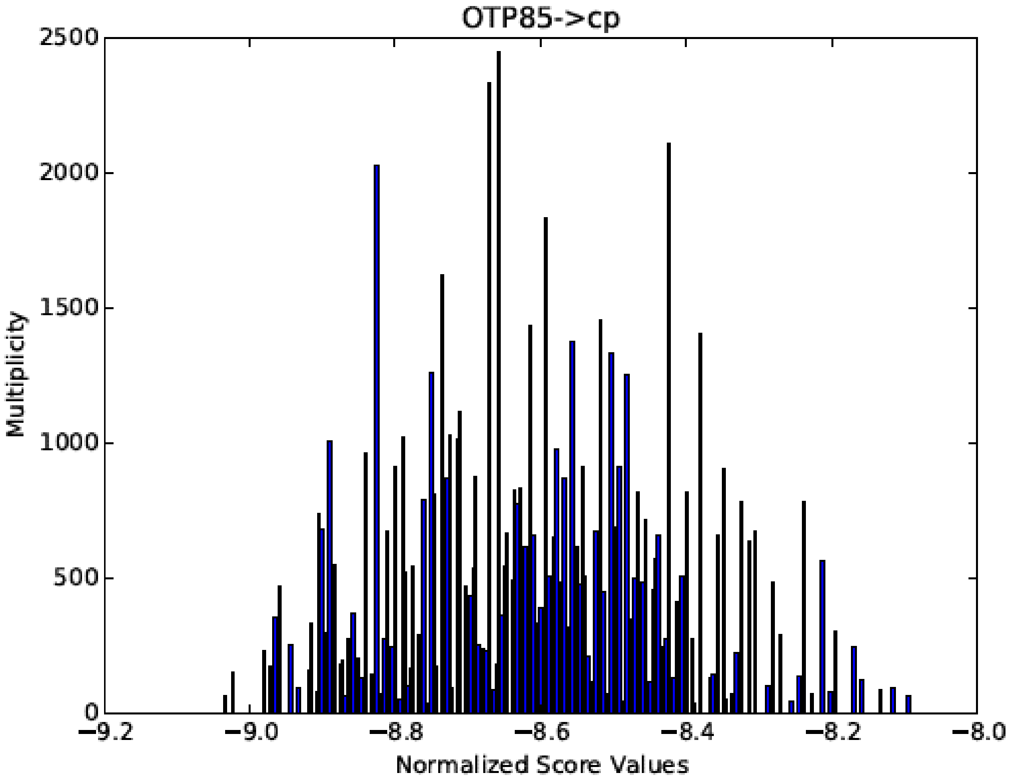
OTP85->cp
Multiplicity
Normalized Score Values
2500
2000
1500
1000
500
0
−9.2
−9.0
−8.8
−8.6
−8.4
−8.2
−8.0

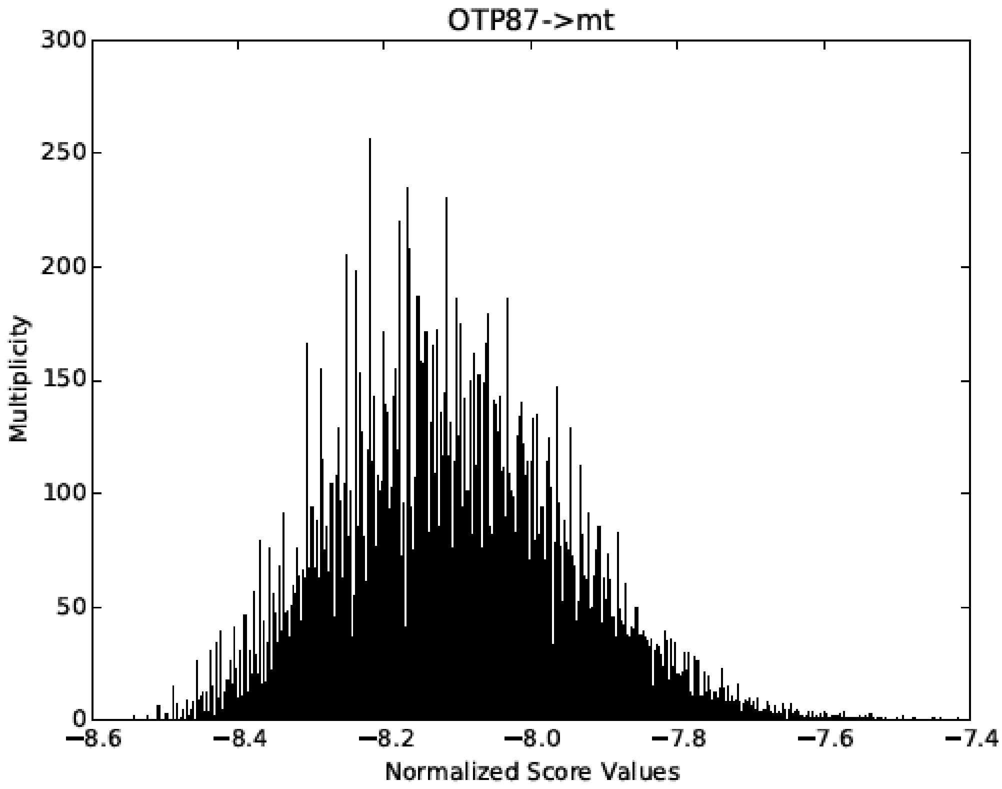
OTP87->mt
Multiplicity
Normalized Score Values
300
250
200
150
100
50
0
−8.6
−8.4
−8.2
−8.0
−7.8
−7.6
−7.4

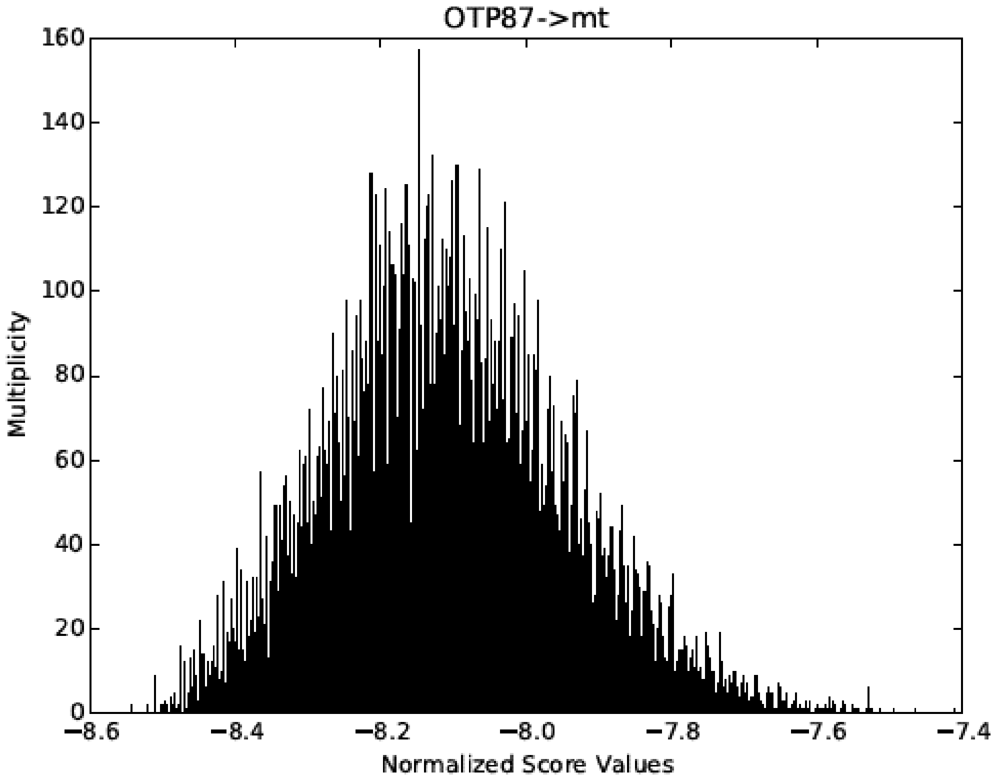
OTP87->mt
Multiplicity
Normalized Score Values
160
140
120
100
80
60
40
20
0
−8.6
−8.4
−8.2
−8.0
−7.8
−7.6
−7.4

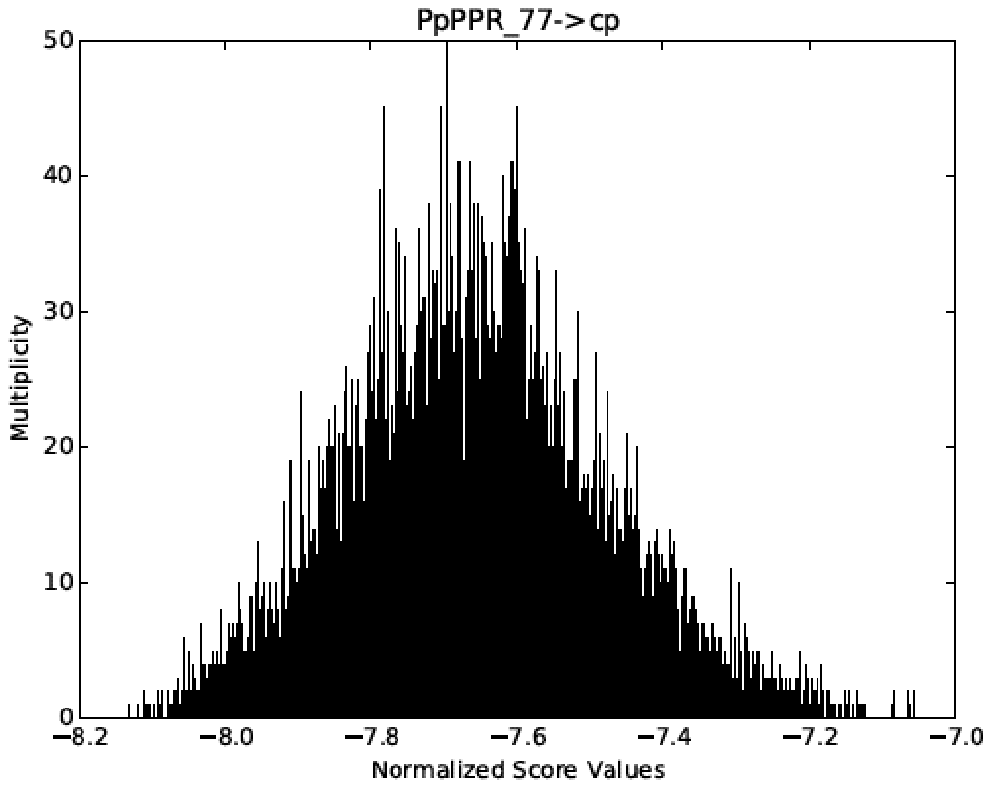
PpPPR_77->cp
Multiplicity
Normalized Score Values
50
40
30
20
10
0
−8.2
−8.0
−7.8
−7.6
−7.4
−7.2
−7.0
